# Impaired local intrinsic immunity to SARS-CoV-2 infection in severe COVID-19

**DOI:** 10.1016/j.cell.2021.07.023

**Published:** 2021-09-02

**Authors:** Carly G.K. Ziegler, Vincent N. Miao, Anna H. Owings, Andrew W. Navia, Ying Tang, Joshua D. Bromley, Peter Lotfy, Meredith Sloan, Hannah Laird, Haley B. Williams, Micayla George, Riley S. Drake, Taylor Christian, Adam Parker, Campbell B. Sindel, Molly W. Burger, Yilianys Pride, Mohammad Hasan, George E. Abraham, Michal Senitko, Tanya O. Robinson, Alex K. Shalek, Sarah C. Glover, Bruce H. Horwitz, Jose Ordovas-Montanes

**Affiliations:** 1 Program in Health Sciences & Technology, Harvard Medical School & MIT, Boston, MA 02115, USA; 2 Ragon Institute of MGH, MIT, and Harvard, Cambridge, MA 02139, USA; 3 Broad Institute of MIT and Harvard, Cambridge, MA 02142, USA; 4 Harvard Graduate Program in Biophysics, Harvard University, Cambridge, MA 02138, USA; 5 Institute for Medical Engineering & Science, Massachusetts Institute of Technology, Cambridge, MA 02139, USA; 6 Department of Medicine, University of Mississippi Medical Center, Jackson, MS 39216, USA; 7 Department of Chemistry, Massachusetts Institute of Technology, Cambridge, MA 02139, USA; 8 Division of Gastroenterology, Hepatology, and Nutrition, Boston Children’s Hospital, Boston, MA 02115, USA; 9 Department of Microbiology, Massachusetts Institute of Technology, Cambridge, MA 02139, USA; 10 Division of Digestive Diseases, University of Mississippi Medical Center, Jackson, MS 39216, USA; 11 Division of Pulmonary, Critical Care, and Sleep Medicine, University of Mississippi Medical Center, Jackson, MS 39216, USA; 12 Obstetrics and Gynecology, University of Mississippi Medical Center, Jackson, MS 39216, USA; 13 Koch Institute for Integrative Cancer Research, Massachusetts Institute of Technology, Cambridge, MA 02139, USA; 14 Division of Emergency Medicine, Boston Children’s Hospital, Boston, MA 02115, USA; 15 Program in Immunology, Harvard Medical School, Boston, MA 02115, USA; 16 Harvard Stem Cell Institute, Cambridge, MA 02138, USA

**Keywords:** SARS-CoV-2, COVID-19, human, nasal mucosa, epithelial immunity, correlates of immunity, interferon, anti-viral, scRNA-seq

## Abstract

SARS-CoV-2 infection can cause severe respiratory COVID-19. However, many individuals present with isolated upper respiratory symptoms, suggesting potential to constrain viral pathology to the nasopharynx. Which cells SARS-CoV-2 primarily targets and how infection influences the respiratory epithelium remains incompletely understood. We performed scRNA-seq on nasopharyngeal swabs from 58 healthy and COVID-19 participants. During COVID-19, we observe expansion of secretory, loss of ciliated, and epithelial cell repopulation via deuterosomal cell expansion. In mild and moderate COVID-19, epithelial cells express anti-viral/interferon-responsive genes, while cells in severe COVID-19 have muted anti-viral responses despite equivalent viral loads. SARS-CoV-2 RNA^+^ host-target cells are highly heterogenous, including developing ciliated, interferon-responsive ciliated, *AZGP1*^high^ goblet, and *KRT13*^*+*^ “hillock”-like cells, and we identify genes associated with susceptibility, resistance, or infection response. Our study defines protective and detrimental responses to SARS-CoV-2, the direct viral targets of infection, and suggests that failed nasal epithelial anti-viral immunity may underlie and precede severe COVID-19.

## Highlights

• scRNA-seq on nasopharyngeal swabs of 58 COVID-19 and healthy participants
• SARS-CoV-2 induces ciliated cell loss with secretory and deuterosomal expansion
• Early, muted anti-viral responses in nasal epithelia in severe COVID-19
• Host-virus co-detection maps cell tropism and intrinsic responses to SARS-CoV-2

## Graphical abstract

**Figure:**
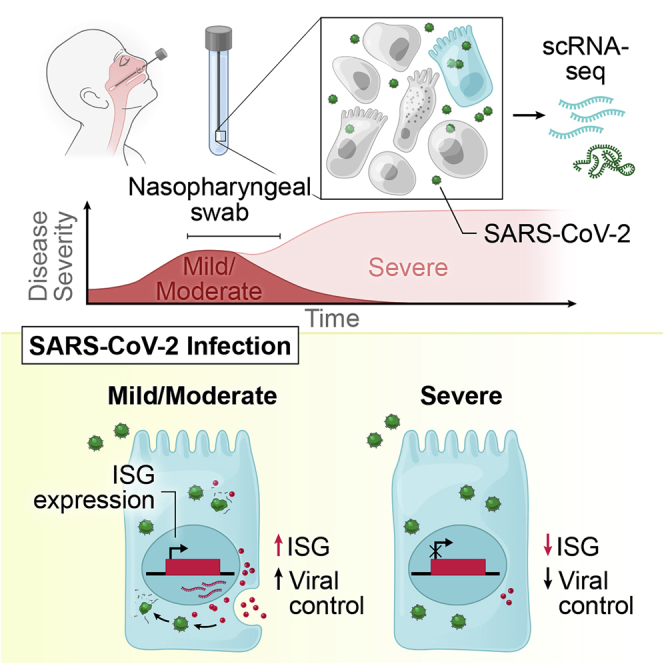

## Teaser

A study of nasopharyngeal swabs from healthy and COVID-19-infected individuals shows how infection leads to compositional changes in the respiratory epithelium, with early dampened antiviral responses in the nasal epithelia likely underlying and preceding severe disease.

## Introduction

The novel coronavirus SARS-CoV-2 emerged in late 2019 and has led to one of the most devastating global pandemics in modern history. Similar to other successful respiratory viruses, high replication within the nasopharynx (Pan et al., 2020; Sanche et al., 2020) and viral shedding by asymptomatic or presymptomatic individuals contributes to enhanced transmissibility (Fears et al., 2020; Meyerowitz et al., 2021) and rapid community spread (Arons et al., 2020; Sakurai et al., 2020; Wang et al., 2020c). COVID-19, the disease caused by SARS-CoV-2 infection, occurs in a fraction of those infected and can carry profound morbidity and mortality. The clinical pictures of COVID-19 vary widely—from a few mild symptoms to prolonged and severe disease characterized by pneumonia, acute respiratory distress syndrome, and diverse systemic effects impacting various tissues (Guan et al., 2020; Huang et al., 2020a). To facilitate effective prophylactics and therapeutics for COVID-19, differentiating protective host mechanisms that support rapid viral clearance and limit disease from those that drive severe and fatal outcomes is essential.

SARS-CoV-2, like other respiratory coronaviruses, enters through the mouth or nares and initially replicates within epithelial cells of the human nasopharynx, generating an upper respiratory infection over several days (Frieman and Baric, 2008; Harrison et al., 2020). A subset of patients develop symptoms of lower respiratory infection, where a combination of inflammatory immune responses and direct viral-mediated pathogenesis can lead to diffuse damage to distal airways, alveoli, and vasculature (Ackermann et al., 2020; Borczuk et al., 2020). Reproducible immune correlates of severe COVID-19 include prolonged detection of proinflammatory cytokines such as IL-6, TNFα, and IL-8, diminished type I and type III interferon, and marked lymphopenia, as well as mixed evidence for immune exhaustion and dysfunctional myeloid populations (Galani et al., 2021; Hadjadj et al., 2020; Kusnadi et al., 2021; Liu et al., 2021; Lucas et al., 2020; Mathew et al., 2020; Mudd et al., 2020; Schulte-Schrepping et al., 2020; Stephenson et al., 2021; Su et al., 2020; Wilk et al., 2020). Most reports have measured host responses in peripheral blood, which may only partially reflect immune status within virally targeted tissues (Ren et al., 2021; Szabo et al., 2020; Weisberg et al., 2021).

Central to understanding SARS-CoV-2-induced disease pathology is identifying the direct cellular targets of infection within human respiratory tissues. Multiple meta-analyses of single-cell RNA-sequencing (scRNA-seq) datasets have nominated putative SARS-CoV-2 targets within the oropharyngeal, nasal, and upper airway tissues, including subsets of ciliated, secretory, and goblet cells, and within the lung parenchyma, type II pneumocytes (Huang et al., 2020b; Lukassen et al., 2020; Muus et al., 2020; Sungnak et al., 2020; Ziegler et al., 2020). A study jointly collecting nasopharyngeal (NP) and bronchoalveolar lavage (BAL) samples from a cohort of COVID-19 patients identified rare SARS-CoV-2 RNA-containing cells assigned to ciliated and secretory cell types (Chua et al., 2020). Further work using human tissues at autopsy found infected ciliated cells lining the trachea and distal lung airways (Hou et al., 2020; Schaefer et al., 2020; Zhu et al., 2020). However, the early targets for SARS-CoV-2 in the nasopharynx, the scope of potential host cells, and the variance in viral tropism across patients and disease courses have yet to be defined.

Compared to other common respiratory viruses, SARS-CoV-2 appears to elicit poor type I interferon (IFN) responses in cultured human epithelial cells, and instead skews toward proinflammatory cytokine profiles, in line with observations from human peripheral studies (Blanco-Melo et al., 2020; Galani et al., 2021; Ravindra et al., 2021). Though animal models have offered critical insight into SARS-CoV-2 behavior *in vivo*, different models vary widely in the severity of SARS-CoV-2-driven disease and associated immunopathology, and incompletely reflect the diversity of viral infection outcomes and natural immune responses in humans (Chandrashekar et al., 2020; Israelow et al., 2020; Muñoz-Fontela et al., 2020; Speranza et al., 2021). Work leveraging human cohorts has identified an enrichment for both inborn errors of type I IFN signaling and the presence of autoantibodies against type I IFNs among patients with severe COVID-19, providing potential explanations for failed or insufficient anti-viral immunity within a subset of severe cases, and further supporting the need for human cohort studies that represent the breadth of host-viral interactions (Bastard et al., 2020, 2021; Combes et al., 2021; Wang et al., 2021a; van der Wijst et al., 2021; Zhang et al., 2020).

Here, we present a comprehensive analysis of the cellular phenotypes in the nasal mucosa during early SARS-CoV-2 infection. To achieve this, we developed tissue-handling protocols that enabled high-quality scRNA-seq from frozen NP swabs collected from a large patient cohort (n = 58) at the early stages of clinical presentation, and created a detailed map of epithelial and immune cell diversity. We found that SARS-CoV-2 infection leads to a dramatic loss of mature ciliated cells, which is associated with secretory cell expansion, differentiation, and the accumulation of deuterosomal cell intermediates—potentially involved in the compensatory repopulation of damaged ciliated epithelium. While we observe broad induction of IFN-responsive and anti-viral genes in cells from individuals with mild or moderate COVID-19, severe COVID-19 is characterized by a dramatically blunted IFN response, and mucosal recruitment of highly inflammatory myeloid populations, which represent the primary sources of tissue pro-inflammatory cytokines including *TNF*, *IL1B*, and *CXCL8*. Further, using unbiased whole-transcriptomic amplification, we map not only host cellular RNA, but also cell-associated SARS-CoV-2 RNA, allowing us to trace viral tropism to specific epithelial subsets and identify host pathways linked with susceptibility or resistance to infection. Together, our data suggest that an early failure of intrinsic anti-viral immunity among nasal epithelial cells responding to SARS-CoV-2 infection may underlie and predict progression to severe COVID-19.

## Results

### Defining cellular diversity in the human nasopharyngeal mucosa

**Figure S1:**
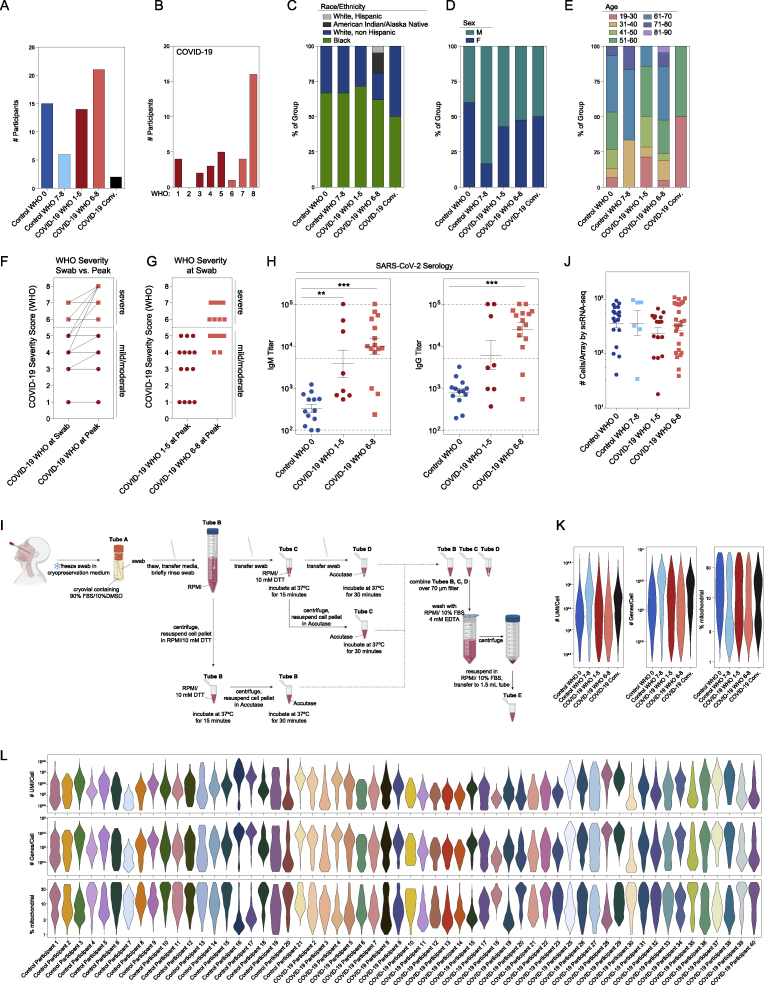
Cohort and cellular composition of nasopharyngeal swabs, related to Figure 1, Table 1 (A–E) Cohort composition and participant demographics (see also Table 1). (A) Number of participants by disease group and peak WHO severity score. Dark blue: healthy individuals, Control WHO 0; light blue: Non-COVID-19 ICU/respiratory failure, Control WHO 7-8; red: COVID-19 mild/moderate, COVID-19 WHO 1-5; pink: COVID-19 severe, COVID-19 WHO 6-8; black: recent COVID-19, convalescent. (B) Number of participants by WHO severity score, COVID-19 participants only. (C) Participant race and ethnicity by disease group. (D) Participant sex by disease group. (E) Participant age by disease group (F and G) Comparison of WHO severity at swab and peak. WHO severity score among COVID-19 participants at swab (left) and peak (right) (F). WHO severity at swab (G). Red circles: COVID-19 mild/moderate (WHO 1-5) at peak. Pink squares: COVID-19 severe (WHO 6-8) at peak. (H) SARS-CoV-2 serology: IgM (left) and IgG (right) titers from a subset of Control WHO 0 (blue circles, n = 13) and COVID-19 (red circles, mild/moderate: n = 8; pink squares, severe: n = 15) participants. Plasma samples taken on same day of nasopharyngeal swab. Statistical testing by Kruskal-Wallis test with Dunn’s post hoc testing. Asterisks represent results from Dunn’s test: ^∗∗^p < 0.01, ^∗∗∗^p < 0.001. Dashed lines: lower limit of detection: 100; upper limit of detection: 100,000; positive threshold: 5,000. (I) Detailed schematic of sample preparation and cell processing from nasal swabs (created with BioRender). (J) Number of high-quality cells/array recovered for single-cell RNA-seq by disease group. Statistical testing by Kruskal-Wallis test (p = 0.37) with Dunn’s post hoc testing, all p > 0.05. (K) Single-cell quality metrics by group (after filtering for low-quality cells, see STAR Methods). (L) Single-cell quality metrics by participant (after filtering for low quality cells).

**Figure 1:**
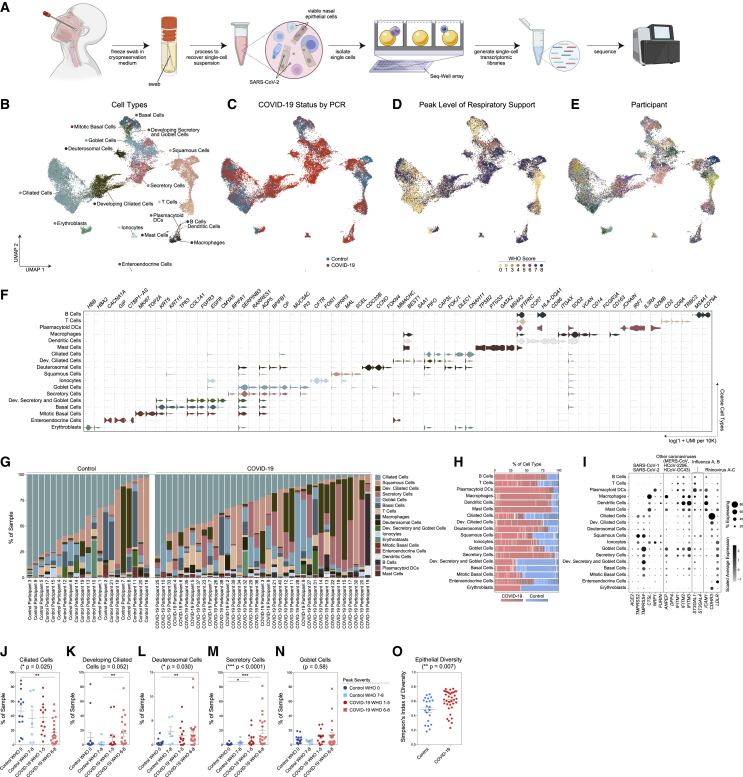
Cellular composition of human nasopharyngeal mucosa (A) Schematic: viable cryopreservation of nasopharyngeal swabs, cellular isolation, and scRNA-seq using Seq-Well S^3^ (created with BioRender.com). (B–E) UMAP of 32,588 cells from all participants, colored by cell type (following iterative Louvain clustering) (B), participant’s COVID-19 status by viral PCR (C), peak level of respiratory support (WHO severity score) (D), and participant (E). (F) Violin plots of cluster marker genes (FDR < 0.01) for coarse cell type annotations (as in B). (G) Proportional abundance of coarse cell types by participant. (H) Proportional abundance of participants by coarse cell types. Red, COVID-19; blue, control. (I) Expression of entry factors for SARS-CoV-2 and other common upper respiratory viruses. Dot size represents fraction of cell type (rows) expressing a given gene (columns). Dot hue represents scaled average expression by gene column. (J–N) Proportion of ciliated cells (J), developing ciliated cells (K), deuterosomal cells (L), secretory cells (M), and goblet cells (N) by sample, separated by peak level of respiratory support. Statistical test above graph represents Kruskal-Wallis test results across all groups (following FDR correction across cell types). Statistical significance asterisks within box represent results from Dunn’s post hoc testing. ^∗^p < 0.05, ^∗∗^p < 0.01, ^∗∗∗^p < 0.001. (O) Simpson’s Diversity index (plotted as 1-D, increasing values represent higher diversity) across epithelial cell types in COVID-19 versus control. Significance by Student’s t test. Lines represent mean ± SEM. See also Figure S1, Table S1.

NP swabs were collected from 58 individuals from the University of Mississippi Medical Center (UMMC) between April and September 2020. This cohort consisted of 35 individuals who had a positive SARS-CoV-2 PCR NP swab on the day of hospital presentation. A control group consisted of 15 individuals who were asymptomatic and had a negative SARS-CoV-2 NP PCR, 6 intubated individuals in the intensive care unit without a recent history of COVID-19 and negative SARS-CoV-2 NP PCR, and 2 additional individuals with recent history of COVID-19 and negative SARS-CoV-2 NP PCR, classified as “convalescent” (Table 1, Figures S1A–S1H, see STAR Methods for full inclusion and exclusion criteria). Using the World Health Organization (WHO) guidelines for stratification and classification of COVID-19 severity, we grouped individuals with COVID-19 based on the maximum (“peak”) level of required respiratory support (World Health Organization, 2020). NP samples were obtained by a trained healthcare provider and rapidly cryopreserved to maintain cellular viability (Figures 1A and S1I). Swabs were processed to recover single-cell suspensions (mean ± SEM: 57,000 ± 15,000 total cells recovered per swab), before generating single-cell transcriptomes using Seq-Well S^3^ (Gierahn et al., 2017; Hughes et al., 2019).

**Table 1:** Participant characteristics

	Control (WHO score 0)	Intubated control (WHO score 7–8)	COVID-19 m/m (WHO score 1–5)	COVID-19 severe (WHO score 6–8)	COVID-19 conv. (WHO score 0)
Case number	25.9% (15/58)	10.3% (6/58)	24.1% (14/58)	36.2 (21/58)	3.4% (2/58)

**Age (years)**					

Minimum	27	33	19	28	20
Median (IQR)	58 (16)	65.5 (31)	49.5 (17.8)	62 (13)	N/A
Maximum	73	71	69	84	57

**Sex**					

Female	60% (9/15)	16.7% (1/6)	42.9% (6/14)	47.6% (10/21)	50% (1/2)
Male	40% (6/15)	83.3% (5/6)	57.1% (8/14)	52.4% (11/21)	50% (1/2)

**Ethnicity**					

Hispanic	0% (0/15)	0% (0/6)	0% (0/14)	4.8% (1/21)	0% (0/2)
Not Hispanic	100% (15/15)	100% (6/6)	100% (14/14)	95.2% (20/21)	100% (2/2)

**Race**					

Black/African American	66.7% (10/15)	66.7% (4/6)	71.4% (10/14)	61.9% (13/21)	50% (1/2)
White	33.3% (5/15)	33.3% (2/6)	28.6% (4/14)	23.8% (5/21)	50% (1/2)
American Indian	0% (0/15)	0% (0/6)	0% (0/14)	14.3% (3/21)	0% (0/2)

**BMI**					

Median (IQR)	37.5 (14.4)	30.5 (18.1)	23.0 (11.6)	31.9 (14.2)	40.7

**Pre-existing conditions**					

Diabetes	40% (6/15)	33.3% (2/6)	28.6% (4/14)	71.4% (15/21)	0% (0/2)
Chronic kidney disease	6.7% (1/15)	0% (0/6)	7.1% (1/14)	19.0% (4/21)	0% (0/2)
Congestive heart failure	6.7% (1/15)	16.7% (1/6)	0% (0/14)	4.8% (1/21)	0% (0/2)
Lung disorder	6.7% (1/15)	16.7% (1/6)	28.6% (4/14)	38.1% (8/21)	0% (0/2)
Hypertension ^∗^	86.7% (13/15)	50% (3/6)	42.9% (6/14)	81.0% (17/21)	0% (0/2)
IBD	13.3% (2/15)	0% (0/6)	0% (0/14)	0% (0/21)	50% (1/2)

**Treatment**					

Corticosteroids	N/A	33.3% (2/6)	42.9% (6/14)	66.7% (14/21)	N/A
Remdesivir	N/A	0% (0/6)	14.3% (2/14)	4.8% (1/21)	N/A
28-day mortality ^∗∗∗^	0% (0/15)	33.3% (2/6)	0% (0/14)	76.2% (16/21)	0% (0/2)

Continuous variables were compared by Kruskal-Wallis test. Categorical variables were compared by chi-square test. ^∗∗∗^p < 0.001, ^∗^p < 0.05, otherwise non-significant. m/m, mild/moderate; conv, convalescent; IQR, inter-quartile range; BMI, body mass index; IBD, inflammatory bowel disease.

**Figure S2:**
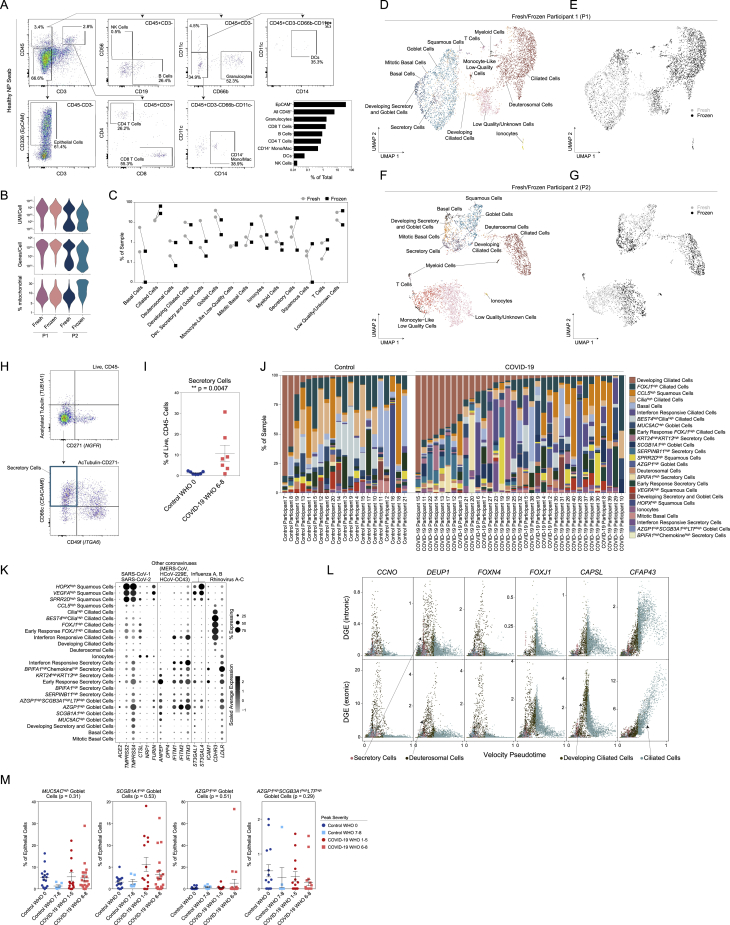
Epithelial diversity and differentiation in the nasopharyngeal mucosa during COVID-19, related to Figure 2 (A) Flow cytometry and gating scheme of immune cells from a fresh nasopharyngeal (NP) swab. Representative healthy participant. Bottom right: quantification of cellular proportions. (B) Quality metrics for matched fresh versus frozen NP swabs from two healthy participants (P1 and P2). (C) Percent composition of each cell type by processing type: fresh (gray circles) or frozen (black squares). (D and E) UMAP of cells from P1, colored by cell types (D) and fresh (gray) versus frozen (black) (E). (F and G) UMAP of cells from P2, colored by cell types (F) and fresh (gray) versus frozen (black) (G). (H) Flow cytometry and gating scheme of epithelial cells from an NP swab. Representative data from a participant with severe COVID-19. (I) Secretory cell proportion of live, CD45- cells from NP swabs. Healthy donors (Control WHO 0): n = 7. Severe COVID-19 (COVID-19 WHO 6-8): n = 7. Secretory cells identified as Live, CD45^-^ATubulin^-^CD271^-^CD49f^-^CD66c^+^ cells. Statistical testing: Wilcoxon signed-rank test: ^∗∗^p = 0.0047. (J) Proportional abundance of detailed epithelial cell types by participant. Ordered within group by developing ciliated cell proportion. (K) Expression of entry factors for SARS-CoV-2 and other common upper respiratory viruses among detailed epithelial cell types. Dot size represents fraction of cell type (rows) expressing a given gene (columns). Dot hue represents scaled average expression by gene column. (L) Plot of gene expression by epithelial cell velocity pseudotime (over all epithelial cells). Select genes significantly associated with ciliated cell pseudotime (FDR < 0.01). Points colored by coarse cell type annotations. Top: alignment to unspliced (intronic) regions. Bottom: alignment to spliced (exonic) regions. (M) Proportion of goblet cell subtypes (detailed annotation) by sample, normalized to all epithelial cells. Statistical test above graph represents Kruskal-Wallis test results across all groups (following FDR correction).

Among all COVID-19 and control samples, we recovered 32,871 genes across 32,588 cells (following filtering and quality control) and annotated 18 clusters corresponding to distinct cell types across immune and epithelial identities (Figures 1B–1E and S1J–S1L, Table S1). We individually annotated clusters based on several references (Deprez et al., 2020; Garcıá et al., 2019; Ordovas-Montanes et al., 2018). Among epithelial cell types, we identified basal cells by their expression of *TP63, KRT15,* and *KRT5*, and mitotic basal cells using genes involved in the cell cycle (*MKI67, TOP2A)* (Figure 1F). We resolved large populations of secretory cells and goblet cells (*KRT7*, *CXCL17, F3, AQP5, CP)*; despite strong transcriptional similarity, we distinguished between goblet and secretory cells based on expression of *MUC5AC-*expressing goblet, and *BPIFA1-*expressing secretory cells. We also resolved a population of ionocytes (*FOXI1, FOXI2*, *CFTR)*, a recently identified specialized subtype of secretory cell involved in regulating mucus viscosity within respiratory epithelia (Montoro et al., 2018; Plasschaert et al., 2018). Squamous cells were identified by expression of *SCEL*, as well as multiple SPRR genes, and potentially derive from the squamous epithelium of the anterior nose or posterior pharynx. Ciliated cells (*FOXJ1* and a ciliogenesis gene program, e.g., *DLEC1, DNAH11, CFAP43*) were the most numerous epithelial cell type recovered. We also identified two populations of precursor ciliated cells: one, termed “developing ciliated cells,” expressed canonical ciliated cell genes such as *FOXJ1*, *CAPSL*, and *PIFO* at lower levels than mature ciliated cells and lacked expression of cilia-forming genes; we also resolved deuterosomal cells (*DEUP1, CCNO, CDC20B, FOXN4, HES6*)—a ciliated cell precursor arising from secretory cell/goblet cell differentiation (Garcıá et al., 2019). Among lymphoid cells, we recovered T cells (*CD3E, CD2, TRBC2*) and B cells (*MS4A1, CD79A, CD79B*). Among myeloid cell types, we recovered a large population of macrophages (*CD14, FCGR3A, VCAN*), dendritic cells (*CCR7, CD86*), and plasmacytoid DCs (*IRF7, IL3RA*). Relative to true tissue-resident abundances, we under-recovered granulocytes, likely due to the intrinsic fragility of these cell types and the cryopreservation required in our sample pipeline (Figures S2A–S2G). We recovered a small population of mast cells (*GATA2, TPSB2, PTGS2*) (Dwyer et al., 2021). Each cell type is represented by cells from numerous participants. From each participant, we recovered a diversity of cell types and states, though the cellular composition is highly variable between distinct individuals (Figures 1G and 1H).

We interrogated each cell type for the expression of host factors utilized by common respiratory viruses to facilitate cellular entry (Figure 1I; Hoffmann et al., 2020; Li et al., 2003; Sungnak et al., 2020; Wang et al., 2020b; Wrapp et al., 2020; Yan et al., 2020). We find *ACE2* expression highest among secretory cells and goblet cells, and to a lesser extent in ciliated cells, developing ciliated cells, deuterosomal cells, and squamous cells—suggesting that these cells are likely targets for SARS-CoV-2 (and other betacoronaviruses that use ACE2 as their primary cellular entry factor). SARS-CoV-2 spike protein requires “priming” by host proteases such as *TMPRSS2, TMPRSS4, CTSL,* and *FURIN* for effective cell entry (Hoffmann et al., 2020). *TMPRSS2*, likely the principal host factor for SARS-CoV-2 S cleavage, is found in highest abundance in squamous cells, followed by modest expression in all other epithelial cell types. Similarly, *CTSL* (and other cathepsins) is found across diverse epithelial and myeloid cell types.

To assess compositional differences by disease severity, we grouped SARS-CoV-2-positive and SARS-CoV-2-negative participants by their peak level of respiratory support according to the WHO scoring system: control WHO 0 (comprising healthy SARS-CoV-2 PCR-negative participants, n = 15), control WHO 7–8 (SARS-CoV-2 PCR-negative, intubated participants treated in the ICU for non-COVID-19 diagnoses, n = 6), COVID-19 WHO 1–5 (SARS-CoV-2 PCR-positive, mild/moderate disease, n = 14), and COVID-19 WHO 6–8 (SARS-CoV-2 PCR-positive, severe disease, n = 21) (Figures 1J–1N). The abundance of ciliated cells is significantly reduced among COVID-19 WHO 6–8 participants compared to healthy controls (Figure 1J) and developing ciliated cells are significantly increased (Figure 1K). Likewise, deuterosomal cells are significantly increased among samples obtained from control WHO 7–8, COVID-19 WHO 1–5, and COVID-19 WHO 6–8 samples (Figure 1L). The percentage of secretory cells is also increased among all COVID-19 participants compared to both the WHO 0 and WHO 7–8 control groups (Figure 1M). We confirmed expansion of secretory cells during severe COVID-19 by flow cytometry in a separate cohort of control WHO 0 (n = 7) and COVID-19 WHO 6–8 (n = 7) participants (Figures S2H and S2I; STAR Methods). Expansion of secretory cells and loss of ciliated cells results in a net gain in epithelial diversity (Figure 1O).

### Epithelial diversity and remodeling after SARS-CoV-2 infection

**Figure 2:**
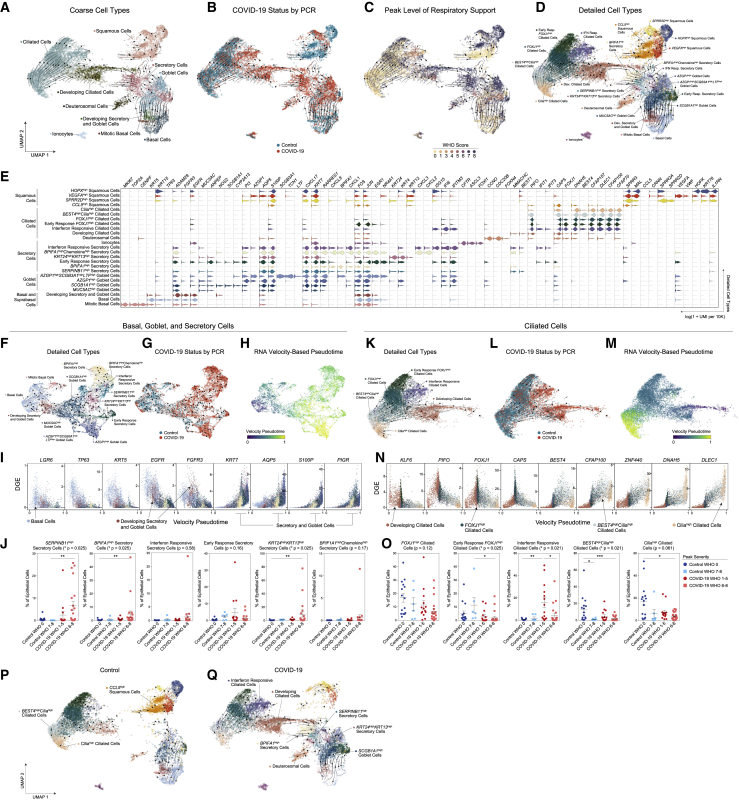
Altered epithelial cell composition in the nasopharynx during COVID-19 (A–D) UMAP of 28,948 cells colored by coarse cell types (A), participant’s COVID-19 status by viral PCR (B), peak level of respiratory support (WHO severity score) (C), and detailed cell types (D). Arrows represent smoothed estimate of cellular differentiation trajectories inferred by RNA Velocity. (E) Violin plots of marker genes for detailed epithelial cell types (as in D). (F–H) UMAP of 9,209 basal, goblet, and secretory cells, following sub-clustering and colored by detailed cell types (F), participant’s COVID-19 status by viral PCR (G), and inferred velocity pseudotime (darker blue shades: precursor cells, intense yellow shades: more terminally differentiated cell types) (H). (I) Gene expression by basal, goblet, and secretory cell velocity pseudotime for select genes. Points colored by detailed cell type annotations. (J) Proportion of secretory cell subtypes by sample, normalized to all epithelial cells. Statistical test above graph represents Kruskal-Wallis test results across all groups (following FDR correction). Statistical significance asterisks within box represent results from Dunn’s post hoc testing. ^∗^p < 0.05, ^∗∗^p < 0.01, ^∗∗∗^p < 0.001. Lines represent mean and SEM. (K–M) UMAP of 13,913 ciliated cells, following sub-clustering and colored by detailed cell types (K), participant’s COVID-19 status by viral PCR (L), and inferred velocity pseudotime (darker blue shades, precursor cells; intense yellow shades, more terminally differentiated cell types) (M). (N) Gene expression by ciliated cell velocity pseudotime for select genes. Points colored by detailed cell type annotations. (O) Proportion of ciliated cell subtypes by sample, normalized to all epithelial cells. (P and Q) UMAP as in (A), separated by only control participants (P, 13,210 epithelial cells) or COVID-19 participants (Q, 15,738 epithelial cells). See also Figure S2, Table S1.

Next, we sought to more completely delineate the diversity of epithelial cells through iterative clustering and sub-clustering (see STAR Methods, Figures 2A–2E and S2J, Table S1). We examined epithelial subtypes for their expression of host entry factors which facilitate viral entry among common upper respiratory pathogens (Figure S2K). Among goblet cells, *AZGP1*^high^ goblet cells express the highest abundance of *ACE2* mRNA, suggesting this cell type may be a preferential target for SARS-CoV-2. Likewise, early response secretory cells, *KRT24*^high^*KRT13*^high^ secretory cells, and interferon responsive secretory cells all express elevated abundances of *ACE2*. To map the differentiation and inter-relationships between epithelial cell types, we applied single-cell RNA velocity (scVelo), which leverages RNA splicing dynamics to infer developmental trajectories (STAR Methods; Bergen et al., 2020; La Manno et al., 2018). Globally, RNA velocity appropriately places basal cells and mitotic basal cells as the “root” of cellular transitions, which then progresses through developing secretory and goblet cells to secretory and goblet cells. Developing ciliated cells and ciliated cells are placed “later” in the differentiation trajectory, distal to development of both secretory and deuterosomal cells, consistent with current models where ciliated cells represent a terminally differentiated state and may arise from these precursor cell types (Garcıá et al., 2019). Together, this analysis enables us to map the developmental relationships between major epithelial cell compartments and connect the loss of “terminally differentiated” or “mature” cell types in COVID-19, e.g., ciliated cells, with the concurrent expansion of their precursors: secretory, deuterosomal, and developing ciliated cells (Figure S2L).

We next analyzed developmental transitions *among* detailed epithelial cell subtypes. When considering only basal, goblet, and secretory cell subtypes, we found *LGR5, TP63*, *EGFR*, and *KRT5* expression gradually decline across basal and developing secretory and goblet cells, while expression of secretory and goblet cell-specific markers (*KRT7*, *AQP5)* progressively increase (Figures 2F–2I). The majority of secretory and goblet clusters are represented by cells from individuals with positive SARS-CoV-2 PCRs, with significant expansion of *SERPINB11*^high^ secretory cells (representing a “generic” or un-differentiated secretory subtype), *BPIFA1*^high^ secretory cells, and *KRT24*^high^*KRT13*^high^ secretory cells (which resemble KRT13^+^ “hillock” cells) among cells from individuals with severe COVID-19 (Figures 2J and S2M). RNA velocity curves predict multiple routes for development between different secretory and goblet subtypes (Figure 2F), suggesting maintained capacity for differentiation and de-differentiation even among “mature” cell types, consistent with the current understanding of respiratory secretory cell plasticity (Tata et al., 2013).

Ciliated cell subtypes were also analyzed via RNA velocity and pseudotemporal ordering (Figures 2K–2N). The velocity pseudotime predicts progression from developing ciliated cells to *FOXJ1*^high^ ciliated cells, to *BEST4*^high^cilia^high^ ciliated cells, and terminating in cilia^high^ ciliated cells (Figure 2M). IFN-responsive ciliated cells and early response *FOXJ1*^high^ ciliated cells represent phenotypic deviations from this ordered progression, and therefore appear collapsed/unresolved along this trajectory with the same pseudotime range as *FOXJ1*^high^ ciliated cells. Among COVID-19 participants, we observe decreased proportions of both cilia^high^ and *BEST4*^high^cilia^high^ ciliated cells, two subsets which represent the most terminally differentiated ciliated cell subtypes (Figure 2O). This effect is particularly pronounced among individuals with severe disease, suggesting that the overall reduction in upper airway ciliated cells during COVID-19 preferentially affects terminally differentiated subsets, potentially due to delayed replenishing from secretory/deuterosomal precursors or enhanced susceptibility to viral-mediated pathogenesis. Among individuals with mild or moderate COVID-19, we find an increase in the proportion of interferon-responsive ciliated cells—averaging 15.9% of all epithelial cells among mild and moderate COVID-19 participants—compared to <1% among healthy controls.

Finally, we directly mapped the developmental transitions among nasal epithelial cells within control (Figure 2P) or COVID-19 participants only (Figure 2Q). Cells from control participants poorly populated the intermediate regions that bridge secretory and goblet cell types to mature ciliated cells (Yoshida et al., 2021). Conversely, regions annotated as multiple secretory cell subsets and developing ciliated cells are uniquely captured from COVID-19 participants. Together, our analysis defines the diversity among cells collected from NP swabs, as well as the nuanced developmental relationships between epithelial cells of the upper airway.

### Alterations to nasal mucosal immune populations in COVID-19

**Figure S3:**
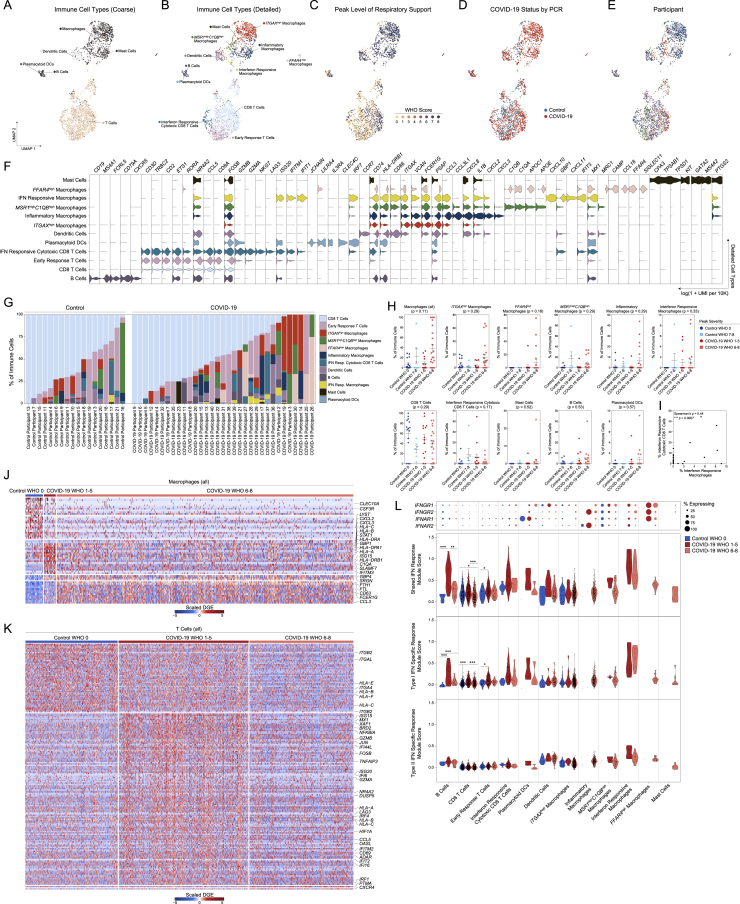
Immune cell diversity in the nasopharyngeal mucosa during COVID-19, related to Figures 1 and 3 (A–E) UMAP of 3,640 immune cells following re-clustering, colored by coarse cell types (A), detailed cell annotations (B), peak level of respiratory support (WHO severity score) (C), participant’s COVID-19 status by viral PCR (D), and participant (E). (F) Violin plots (log(1+normalized UMI per 10k)) of cluster marker genes (FDR < 0.01) for detailed immune cell type annotations (as in B). (G) Proportional abundance of detailed immune cell types by participant. (H) Proportion of immune cell subtypes by sample and disease group, normalized to all immune cells. Statistical test above graph represents Kruskal-Wallis test results across all cell types (following FDR correction). (I) Proportion of interferon responsive macrophages versus proportion of interferon responsive cytotoxic CD8 T cells per sample, normalized to total immune cells. Including all samples, Control and COVID-19 groups. (J and K) Heatmap of significantly DE genes between macrophages (all, coarse annotation) (J) and T cells (all, coarse annotation) (K) from different disease groups. Values represent row(gene)-scaled digital gene expression (DGE) following log(1+UMI per 10K) normalization. (L) Top: Dot plot of *IFNGR1, IFNGR2*, *IFNAR1,* and *IFNAR2* gene expression among all detailed immune subtypes. Bottom: Violin plots of module scores, split by Control WHO 0 (blue), COVID-19 WHO 1-5 (red), and COVID-19 WHO 6-8 (pink). Gene modules represent transcriptional responses of human basal cells from the nasal epithelium following *in vitro* treatment with IFNα or IFNγ. Significance by Wilcoxon signed-rank test. P values following Bonferroni-correction: ^∗^p < 0.05, ^∗∗^p < 0.01, ^∗∗∗^p < 0.001.

As with epithelial cells, we further clustered and annotated detailed immune cell populations (Figure S3, Table S1). Among immune cells, macrophages markedly increase in abundance during severe COVID-19 (Figures S3G and S3H). Multiple specialized myeloid cell types are uniquely detected and enriched among COVID-19 participants, albeit in a subset of participants, and biased to severe COVID-19 cases: *ITGAX*^high^ macrophages, *FFAR*4^high^ macrophages, inflammatory macrophages, and IFN-responsive macrophages (Figure S3H). Rare plasmacytoid DCs and mast cells are recovered as >1% of immune cells only among COVID-19 participants. Finally, we assessed the correlation between distinct immune cell types across all participants. The proportional abundance of dendritic cells, mast cells, and macrophages are highly correlated with one another (p < 0.01), likely indicative of coordinated recruitment during inflammation. IFN-responsive macrophages are correlated with IFN-responsive cytotoxic CD8 T cells (p < 0.01, Figure S3I), suggesting potential direct communication between *IFNG*-expressing tissue-resident T cells and *CXCL9/10/11*-expressing myeloid cells. Collectively, the epithelial and immune compartments are dramatically altered during COVID-19, likely reflecting both protective anti-viral and regenerative responses, as well as pathologic changes underlying progression to severe disease.

### Cell states associated with COVID-19 severity

**Figure S4:**
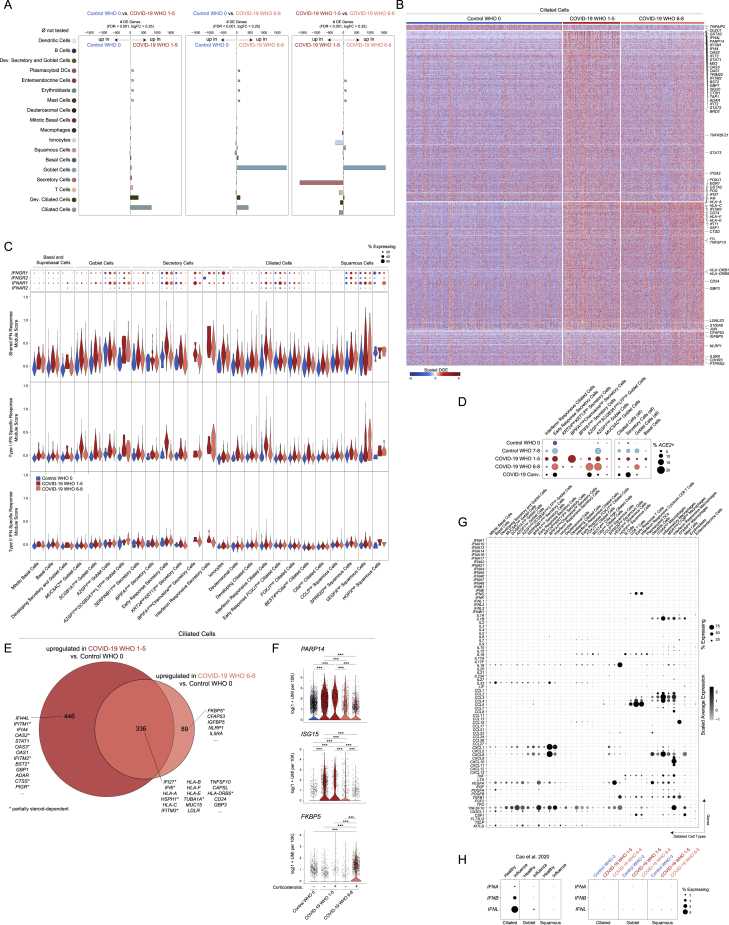
Cell-type-specific and shared transcriptional Responses to SARS-CoV-2 infection, related to Figure 3 (A) Abundance of significant DE genes by coarse cell type between Control WHO 0 and COVID-19 WHO 1-5 samples (left), Control WHO 0 and COVID-19 WHO 6-8 samples (middle) and COVID-19 WHO 1-5 versus COVID-19 WHO 6-8 samples (right). Gene significance cutoffs: FDR-corrected p < 0.001, log2 fold change > 0.25. (B) Heatmap of significantly DE genes between ciliated cells (all, coarse annotation) from different disease groups. Values represent row(gene)-scaled digital gene expression (DGE) following log(1+UMI per 10K) normalization. (C) Top: Dot plot of *IFNGR1, IFNGR2*, *IFNAR1,* and *IFNAR2* gene expression among all detailed epithelial subtypes. Bottom: Violin plots of module scores, split by Control WHO 0 (blue), COVID-19 WHO 1-5 (red), and COVID-19 WHO 6-8 (pink). Gene modules represent transcriptional responses of human basal cells from the nasal epithelium following *in vitro* treatment with IFNα or IFNγ. Significance by Wilcoxon signed-rank test. P values following Bonferroni-correction: ^∗^p < 0.05, ^∗∗^p < 0.01, ^∗∗∗^p < 0.001. (D) Dot plot of *ACE2* expression across select epithelial cell types and subsets. (E) Venn diagram of significantly upregulated genes among ciliated cells between COVID-19 WHO 1-5 versus Control WHO 0 (red) and COVID-19 WHO 6-8 versus Control WHO 0 (pink). Asterisk: genes impacted by corticosteroid treatment within each group. (F) Violin plots of select genes upregulated among ciliated cells in COVID-19 WHO 1-5 participants compared to Control WHO 0 (*PARP14, ISG15*) and in COVID-19 WHO 6-8 participants compared to Control WHO 0 (*FKBP5*). Cells separated by participant treatment with corticosteroids. ^∗∗∗^ FDR-corrected p < 0.001. (G) Dot plot of interferon and cytokine expression among detailed epithelial and immune cell types. (H) Dot plot of type I and type III interferons among ciliated, goblet, and squamous cells. Left: healthy versus influenza A/B virus infected participants from Cao et al., 2020. Right: Control WHO 0 versus COVID-19 WHO 1-5, versus COVID-19 WHO 6-8 participants. Datasets processed and scaled identically.

**Figure 3:**
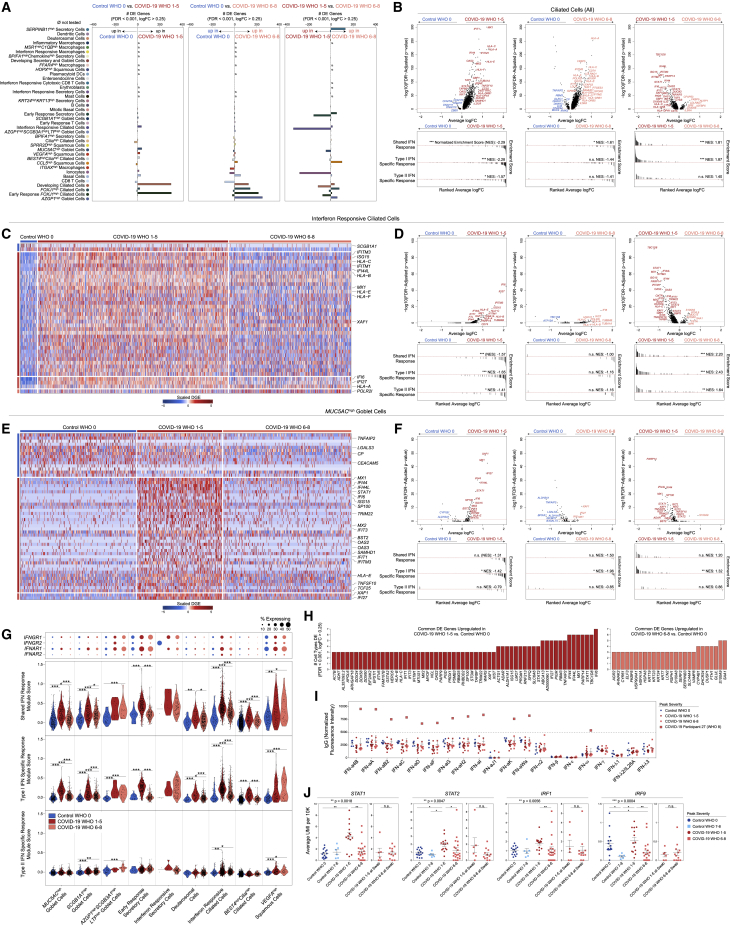
Cell-type-specific and shared transcriptional responses during COVID-19 (A) Abundance of significantly DE genes by detailed cell types between disease groups. FDR-corrected p < 0.001, log2 fold change > 0.25. ø = comparison not tested, too few cells. (B) Top: volcano plots of average log fold change (FC) versus -log_10_(FDR-adjusted p value) for ciliated cells (all, coarse annotation) between disease groups. Horizontal red dashed line: FDR-adjusted p value = 0.05. Bottom: GSEA plots across shared, type I interferon-specific, and type II interferon-specific stimulated genes. Genes ranked by their average log FC between each comparison. Black lines represent the ranked location of genes belonging to the annotated gene set. Bar height represents running enrichment score (NES, normalized enrichment score). p values following Bonferroni-correction: ^∗^p < 0.05, ^∗∗^p < 0.01, ^∗∗∗^p < 0.001. (C) Heatmap of significantly DE genes between interferon responsive ciliated cells from different disease groups. Row(gene)-scaled digital gene expression (DGE) following log(1+UMI per 10K) normalization. (D) Top: Volcano plots related to C for interferon-responsive ciliated cells. Horizontal red dashed line: FDR-adjusted p value = 0.05. Bottom: GSEA plots across shared, type I, and type II interferon-stimulated genes. (E) Heatmap of significantly DE genes between *MUC5AC*^high^ goblet cells from different disease groups. Row(gene)-scaled digital gene expression (DGE) following log(1+UMI per 10K) normalization. (F) Top: Volcano plots related to (E) for *MUC5AC*^high^ goblet cells. Horizontal red dashed line: FDR-adjusted p value = 0.05. Bottom: GSEA plots across shared, type I, and type II interferon-stimulated genes. (G) Top: Dot plot of *IFNGR1, IFNGR2*, *IFNAR1,* and *IFNAR2* gene. Bottom: Violin plots of module scores, split by control WHO 0 (blue), COVID-19 WHO 1–5 (red), and COVID-19 WHO 6–8 (pink). Significance by Wilcoxon signed-rank test. p values following Bonferroni-correction: ^∗^p < 0.05, ^∗∗^p < 0.01, ^∗∗∗^p < 0.001. (H) Common DE genes across detailed cell types. Left (red): COVID-19 WHO 1–5 versus control WHO 0. Right (pink), COVID-19 WHO 6–8 versus control WHO 0. (I) Relative abundances of IgG autoantibodies for human type I, II, and III interferons via multiplexed human antigen microarray (see STAR Methods). Blue circles, control WHO 0, n = 5; red circles, COVID-19 WHO 1–5, n = 12; pink squares, COVID-19 WHO 6–8, n = 8. Large pink squares, autoantibodies against 12 type I interferons from a single donor:,COVID-19 participant 27 (peak WHO severity score: 8, swab WHO severity score: 5). (J) Average expression of *STAT1, STAT2, IRF1,* and *IRF9* among ciliated cells by participant. For each gene: left: participants separated by disease group, determined by participants’ peak WHO severity score. Statistical testing by Kruskal-Wallis test across disease groups (^∗∗^p = 0.0018) with Dunn’s post hoc testing: ^∗^p < 0.05, ^∗∗^p < 0.01, ^∗∗∗^p < 0.001. Right: participants in COVID-19 WHO 6–8 group, separated by level of severity at time of nasal swab. Statistical testing by Wilcoxon signed-rank test, n.s. non-significant, p > 0.05. See also Figures S3 and S4, Tables S2, S3, and S4.

Next, we examined how each cell type responds according to different peak disease severity scores. We performed pairwise differential expression (DE) tests between control WHO 0, COVID-19 WHO 1–5, and COVID-19 WHO 6–8 groups (Tables S2, S3, and S4). Among all coarse cell types, the largest transcriptional changes (measured by the number of DE genes with FDR < 0.001, and log fold change > 0.25) are observed within the epithelial compartment, including ciliated cells, developing ciliated cells, secretory cells, goblet cells, and ionocytes (Figure S4A). Among detailed cell types, we observed the largest transcriptional changes among *AZGP1*^high^ goblet cells, early-response *FOXJ1*^high^ ciliated cells, *FOXJ1*^high^ ciliated cells, *MUC5AC*^high^ goblet cells, *SERPINB11*^high^ secretory cells, early-response secretory cells, and IFN-responsive ciliated cells (Figure 3A). When we directly compared mild or moderate to severe COVID-19, we found that multiple cell types show robust transcriptional changes, most drastically among ciliated cell subtypes (IFN-responsive ciliated cells, *FOXJ1*^high^ ciliated cells, early-response *FOXJ1*^high^ ciliated cells, developing ciliated cells), ionocytes, *SERPINB11*^high^ secretory cells, early-response secretory cells, and *AZGP1*^high^ goblet cells.

Compared to ciliated cells from control WHO 0 participants, cells from both mild/moderate and severe COVID-19 upregulated genes involved in the host response to virus, including *IFI27, IFIT1, IFI6, IFITM3,* and *GBP3*, and both groups induce expression of MHC-I and MHC-II genes (*HLA-A, HLA-C, HLA-F, HLA-E, HLA-DRB1, HLA-DRA*) and other factors involved in antigen processing and presentation (Figures 3B and S4B). Large sets of IFN-responsive and anti-viral genes are exclusively induced among ciliated cells from COVID-19 WHO 1–5 participants when compared to control WHO 0 participants. In a direct comparison of ciliated cells from mild or moderate to severe COVID-19, the cells from individuals with mild or moderate disease show strong upregulation of diverse anti-viral factors, including *IFI44L, STAT1, IFITM1, MX1, IFITM3, OAS1, OAS2, OAS3, STAT2, TAP1, HLA-C, ADAR, XAF1, IRF1, CTSS, CTSB*, and many others. Ciliated cells from severe COVID-19 uniquely upregulate *IL5RA* and *NLRP1*. Together, these DE gene sets suggest exposure to secreted inflammatory factors and type I/II/III IFNs, as well as direct cellular sensing of viral products. Using previously published data from human nasal basal cells treated *in vitro* with either type I (IFNα) or type II (IFNγ) IFNs (Ziegler et al., 2020), we created gene sets that represent the “shared” gene responses to type I and type II IFNs, and the cellular responses specific to either type (Figure 3B). Using gene set enrichment analysis (GSEA), we tested whether the genes that discriminate ciliated cells from different groups (e.g., mild or moderate versus severe COVID-19) imply exposure to specific IFN types. We found that ciliated cells in mild or moderate COVID-19 robustly induce type I IFN-specific gene signatures, both compared to cells from healthy controls as well as from severe COVID-19. Further, when compared to cells from healthy individuals, ciliated cells from individuals with severe COVID-19 did not significantly induce type I or type II IFN-responsive genes, potentially underlying poor control of viral spread.

We next investigated whether these effects were observed among other cell types and subsets. Surprisingly, even *among* cells defined as “IFN-responsive” ciliated cells, cells from mild or moderate COVID-19 participants express higher fold changes of IFN-responsive genes compared to cells from severe COVID-19 participants or healthy controls (Figures 3C and 3D). Other epithelial and immune cell types display a similar pattern: broad IFN-responsive genes (largely type I specific) are strongly upregulated among cells from mild or moderate COVID-19 participants, while cells from severe COVID-19 participants upregulate few shared markers with mild or moderate COVID-19 participants, and instead skew toward inflammatory genes (*S100A8,* S100A9) (Figures 3E–3H, S3J–S3L, and 4C). In some cases, cells from individuals with severe COVID-19 express levels of IFN-responsive or anti-viral genes indistinguishable from healthy controls. Further, the absence of a transcriptional response to secreted IFN cannot be explained by a lack of either IFNα-receptor (*IFNAR1, IFNAR2*) or IFNγ-receptor (*IFNGR1, IFNGR2*) expression. Previous work has identified *ACE2* as among the IFN-induced genes in nasal epithelial cells, with uncertain significance for SARS-CoV-2 infection (Blume et al., 2021; Ng et al., 2020; Onabajo et al., 2020; Ziegler et al., 2020). Indeed, we find modest upregulation of this gene among cells from COVID-19 participants compared to healthy controls. Further, some of the cell subtypes identified as expanded during COVID-19 (e.g., IFN-responsive ciliated cells, *BPIFA1*^high^ secretory cells, *BPIFA1*^high^chemokine^high^ secretory cells, and *KRT24*^high^*KRT13*^high^ secretory cells) express relatively high abundances of *ACE2* (Figure S4D).

**Figure 4:**
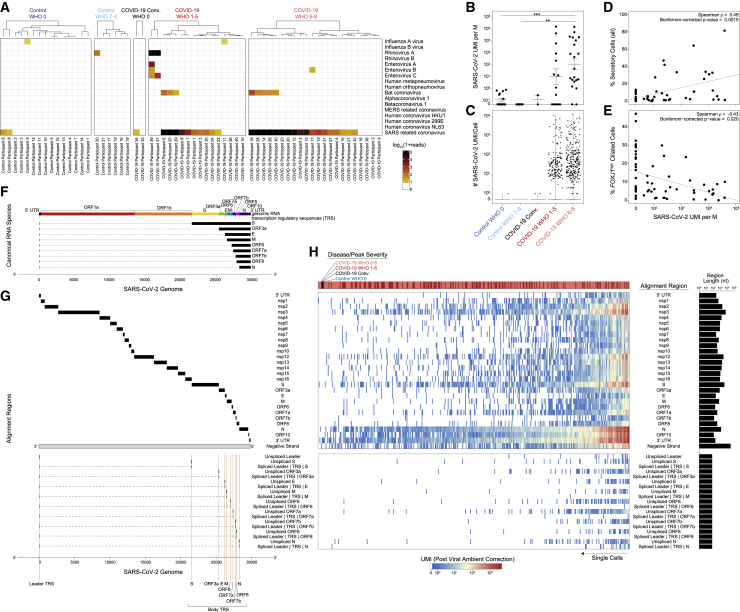
Co-detection of human and SARS-CoV-2 RNA (A) Metatranscriptomic classification of all scRNA-seq reads using Kraken2 (STAR Methods). Results shown from selected respiratory viruses (threshold > 5 reads). (B) Normalized abundance of SARS-CoV-2 aligning UMI from all scRNA-seq reads (including those derived from ambient cell barcodes). p < 0.0001 by Kruskal-Wallis test. Pairwise comparisons using Dunn’s post hoc testing. ^∗∗^p < 0.01, ^∗∗∗^p < 0.001. (C) SARS-CoV-2 UMIs per high-complexity single-cell transcriptome (following correction for ambient viral reads). (D) Proportional abundance of secretory cells (all, coarse annotation) versus total SARS-CoV-2 UMIs (normalized to M total UMIs). (E) Proportional abundance of *FOXJ1*^high^ ciliated cells versus total SARS-CoV-2 UMIs (normalized to M total UMIs). (F) Schematic: SARS-CoV-2 genome and subgenomic RNA species. (G) Schematic: SARS-CoV-2 genomic features annotated in the custom reference genome. (H) Heatmap of SARS-CoV-2 gene expression among SARS-CoV-2 RNA^+^ single cells (following correction for ambient viral reads). Disease group color bar: red, COVID-19 WHO 1–5; pink, COVID-19 WHO 6–8; black, COVID-19 convalescent; blue, control WHO 0. Top heatmap: SARS-CoV-2 genes and regions organized from 5′ to 3′. Bottom heatmap: alignment to 70-mer regions directly surrounding viral TRS sites. See also Figures S5 and S6.

A proportion of COVID-19 participants in our study were concurrently treated with corticosteroids, which mediate broad anti-inflammatory and immunosuppressive effects. For some genes, corticosteroid treatment is associated with a partially suppressed IFN response *within* each group—for instance, ciliated cells from untreated COVID-19 WHO 1–5 participants show higher abundances of *IFITM1, OAS2, IFI6*, and *IFI27* than their corticosteroid-treated counterparts—while still maintaining strong differences in expression *between* severity groups (Figures S4E and S4F). Interestingly, induction of *FKBP5* expression among ciliated cells from severe COVID-19 participants is fully explained by corticosteroid treatment, consistent with the role for this protein in modulating glucocorticoid receptor activity. The majority of anti-viral genes were not impacted by corticosteroid treatment, including *STAT1*, *STAT2, IFI44,* and *ISG15* (Liu et al., 2021). Together, these data demonstrate global blunting of the anti-viral/IFN response among nasopharyngeal epithelial cells during severe COVID-19.

We next attempted to query the source of local IFN. Many tissue-resident immune cells reside principally within the deeper lamina propria and submucosal spaces, and are therefore, as expected, poorly represented in our dataset due to our sampling strategy (swabbing of surface epithelial cells) (Deprez et al., 2020; Ordovas-Montanes et al., 2018). Accordingly, we find few immune cell types producing IFNs: *IFNA* and *IFNB* are absent, rare *IFNL1* reads are observed among T cells and macrophages, and *IFNG* is robustly produced from IFN-responsive cytotoxic CD8 T cells (Figure S4G). We could not detect expression of any IFN types among epithelial cells, which differs dramatically from previous observations of robust type I/III IFN expression among nasal ciliated cells during influenza A and B infection (also captured via Seq-Well S^3^; Cao et al., 2020) (Figure S4H). Rather, we observe robust induction of other inflammatory molecules from immune and epithelial cell types. *CXCL8* is produced by several specialized secretory cell types, including those uniquely expanded in COVID-19. Inflammatory macrophages and IFN-responsive macrophages represent the primary sources of local *TNF*, *IL6*, and *IL10*, and uniquely express high abundances of chemoattractant molecules such as *CCL3, CCL2,* and *CXCL8.* Interestingly, IFN-responsive macrophages appear to be a principal source of *CXCL9, CXCL10,* and *CXCL11* (Figure S4G).

We directly tested whether the lack of an IFN-stimulated response among nasal epithelial cells in severe COVID-19 participants could be explained by autoantibody-mediated inhibition of secreted interferons as reported in other cohorts (Bastard et al., 2020, 2021; Wang et al., 2021a). Using matched plasma collected at the time of NP swab, we analyzed a subset of 25 participants for IgG and IgM antibodies targeting a large panel of potential antigens (using a microarray-based antibody hybridization platform; see STAR Methods). Here we found evidence for IgG autoantibodies targeting IFN-ω and 11 IFNα subtypes in 1/8 participants who developed severe COVID-19, 0/12 participants with mild or moderate disease, and 0/5 healthy donors (Figure 3I). We caution against generalizing this result due to our limited cohort size; we note, however, that our findings agree well with the expected proportion (∼10%) of severe individuals with autoantibodies to IFN components from published data (Bastard et al., 2020).

To better understand participant-to-participant variability in anti-viral and IFN-responsive gene signatures, we analyzed the average expression of *STAT1, STAT2, IRF1,* and *IRF9*—key transcription factors responsible for the induction of IFN-stimulated gene expression and IFN-induced genes themselves—among ciliated cells from each participant (Figure 3J). We found that the expression of *STAT1*, *STAT2*, and *IRF1* was indistinguishable among cells from control WHO 0, control WHO 7–8, and COVID-19 WHO 6–8 participants. *IRF9* was diminished among COVID-19 WHO 6–8 participants and control WHO 7–8 participants compared to healthy donors and participants with mild or moderate COVID-19. Intriguingly, despite the absence of autoantibodies directed at type I interferons, nearly all participants who developed severe COVID-19 failed to induce *STAT1, STAT2, IRF1,* and *IRF9* expression (among other IFN-stimulated genes). Even individuals who had milder disease and limited requirement for respiratory support at the time of nasal swab, but later went on to develop severe or fatal COVID-19 (swab WHO 1–5, peak WHO 6–8), already had diminished *STAT1* expression at the time of nasal swab (Figure 3J). This suggests a potential predictive value of poor interferon-stimulated gene (ISG) induction.

### Co-detection of viral and host RNA and correlates of nasal viral load

**Figure S5:**
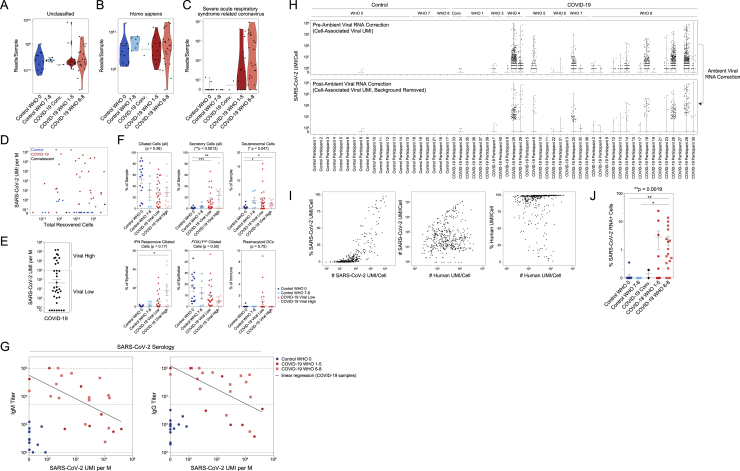
Detection of SARS-CoV-2 RNA from single-cell RNA-seq data, related to Figures 4 and 5 (A–C) Metatranscriptomic classification of all scRNA-seq reads using Kraken2. Reads per sample annotated as unclassified (A), *Homo sapiens* (B), SARS-related coronaviruses (C). (D) Total recovered cells per sample versus normalized abundance of SARS-CoV-2 aligning UMI from all scRNA-seq UMI (including those derived from ambient/low-complexity cell barcodes). (E) Normalized abundance of SARS-CoV-2 aligning UMI across all COVID-19 participants. Dashed line represents partition between “Viral High” versus “Viral Low” samples (1,000 SARS-CoV-2 UMI/million (M) UMI). (F) Proportional abundance of selected cell types according to total SARS-CoV-2 abundance among COVID-19 samples, stratified by cutoffs in panel E. Statistical test above graph represents FDR-corrected Kruskal-Wallis test statistic across all groups. Statistical significance asterisks within box represent significant results from Dunn’s post hoc testing. ^∗^p < 0.05, ^∗∗^p < 0.01, ^∗∗∗^p < 0.001. (G) Normalized abundance of SARS-CoV-2 aligning UMI versus anti-SARS-CoV-2 IgM (left) or IgG titers (right). Plasma samples taken on same day of nasopharyngeal swab. Subset of Control WHO 0 (blue circles, n = 13) and COVID-19 (red circles, mild/moderate: n = 8; pink squares, severe: n = 15) participants. Dashed lines: lower limit of detection: 100; upper limit of detection: 100,000; positive threshold: 5,000. Pearson’s correlation of COVID-19 samples: IgM: r = −0.59, ^∗∗^p = 0.0028; IgG: r = −0.60, ^∗∗^p = 0.0025. (H) Abundance of SARS-CoV-2 aligning UMI/cell by participant prior to (top) and following (bottom) ambient viral RNA correction (see STAR Methods). (I) Quality metrics among 415 SARS-CoV-2 RNA^+^ cells (associated with high-quality cell barcodes and following ambient viral RNA correction). Left: abundance of SARS-CoV-2 aligning UMI versus percent of all SARS-CoV-2 aligned reads (per cell barcode). Middle: abundance of human (GRCh38)-aligning UMI versus abundance of SARS-CoV-2 aligning UMI. Right: abundance of human (GRCh38) aligning UMI versus percent of all human aligned reads (per cell barcode). (J) Percent SARS-CoV-2 RNA^+^ cells (associated with high-quality cell barcodes and following ambient viral RNA correction) per donor, separated by disease group. Statistical test above graph represents Kruskal-Wallis test statistic across all groups. Statistical significance asterisks within box represent significant results from Dunn’s post hoc testing. ^∗^p < 0.05, ^∗∗^p < 0.01.

We next tested whether the observed epithelial and immune phenotypes were associated with altered local viral abundance. To perform an unbiased search for co-detected viral, bacterial, and fungal genomic material, we used metatranscriptomic classification to assign reads according to a comprehensive reference database (previously described, see STAR Methods, Lemieux et al., 2021; Wood et al., 2019). As expected, the majority (28/38) of swabs from individuals with COVID-19 contain reads classified as SARS coronavirus species (Figures 4A and S5A–S5C). Among samples containing SARS coronavirus genomic material, the read abundance ranged from 2e0 to 8.8e6 reads (1.8e−3 to 1.9e4 reads/million [M] total reads). We found little evidence for co-occurring respiratory viruses.

Next, we analyzed all SARS-CoV-2-aligned unique molecular identifiers (UMIs) following alignment to a joint genome containing both human and SARS-CoV-2 (Kim et al., 2020). We took the sum of all SARS-CoV-2 aligning UMIs from a given participant—both associated with high-complexity single-cell transcriptomes and ambient RNA—as a representative measure of the total SARS-CoV-2 burden within the tissue microenvironment. As observed using metatranscriptomic classification, we found relatively low/spurious alignments to SARS-CoV-2 among control participants, while swabs from COVID-19 participants contained a wide range of SARS-CoV-2 reads (Figures 4B, 4C, S5D, and S5E). SARS-CoV-2 UMIs were detected in 80% (28/35) of COVID-19 participants. Samples from participants who developed severe COVID-19 contained significantly higher abundances of SARS-CoV-2 aligning UMIs than both control groups, with an average of 1.1e2 ± 2.8e0 (geometric mean ± SEM) UMIs per million (M) aligned UMIs (ranging from 0 to 1.5e5 per sample); swabs from participants with mild or moderate COVID-19 contained slightly fewer SARS-CoV-2 aligning UMIs, with an average of 1.1e1 ± 4.3e0 UMIs per M. Among all cell types, we observe that secretory cells are significantly positively correlated with the total viral abundance (Spearman’s rho = 0.49, Bonferroni-corrected p = 0.0015), while *FOXJ1*^high^ ciliated cells are negatively correlated (Spearman’s rho = −0.43, Bonferroni-corrected p = 0.020, Figures 4D and 4E). We binned the samples from COVID-19 participants into “viral low” and “viral high” groupings (based on an arbitrary cutoff of 1e3 SARS-CoV-2 UMIs per M; robust to a range of partition choices, Figures S5E and S5F). IFN-responsive ciliated cells are expanded among “viral high” COVID-19 samples, and plasmacytoid DCs are only found in “viral low” samples. Finally, in a subset of patients for whom we obtained matched plasma samples on the same day of NP swab (n = 36), we observe SARS-CoV-2 UMI abundance is inversely correlated with the SARS-CoV-2 IgM and IgG titers (Figure S5G). As severe COVID-19 has been shown to correlate with higher antibody titers, this suggests that several individuals in our cohort are sampled early in their disease trajectory, though we note there is substantial complexity in interpreting how antibody levels align with the timing of infection, viral load, and ISGs (Garcia-Beltran et al., 2021; Long et al., 2020; Zohar et al., 2020).

### Cellular targets of SARS-CoV-2 infection in the nasopharynx

Next, we aimed to differentiate SARS-CoV-2 UMIs derived from ambient or low-complexity cell barcodes from those likely reflecting intracellular RNA molecules within high-complexity single-cell transcriptomes (Cao et al., 2020; Delorey et al., 2021; Fleming et al., 2019; Kotliar et al., 2020). We filtered to viral UMIs associated with cells presented in Figure 1, removing those associated with low-complexity or ambient-only cell barcodes (Figure S5H). Next, we estimated the proportion of ambient RNA contamination per single cell and the abundance of SARS-CoV-2 RNA within the extracellular/ambient environment (i.e., not cell-associated) per sample. Using these parameters, we tested whether the amount of viral RNA associated with a given single-cell transcriptome was significantly higher than expected from ambient spillover. This enabled us to identify cell barcodes whose SARS-CoV-2-aligning UMIs were likely driven by spurious contamination, and annotate single cells that contain probable cell-associated or intracellular SARS-CoV-2 RNA (Figures 4C and S5H). Across all single cells, we recover 415 high-confidence SARS-CoV-2 RNA^+^ cells across 21 participants, which we confirmed is not driven by technical factors (Figure S5I). 262 SARS-CoV-2 RNA^+^ cells are from participants who developed severe COVID-19 and 150 from mild or moderate COVID-19. We detect three SARS-CoV-2 RNA^+^ cells from participants with negative SARS-CoV-2 PCR: two from a participant identified as “COVID-19 convalescent,” and one from a control participant. Among participants with any SARS-CoV-2 RNA^+^ cells, we detect 20 ± 7 (mean ± SEM) SARS-CoV-2 RNA^+^ cells per sample (range 1–119), amounting to 4% ± 1.3% (range 0.1%–24%) of the total recovered cells per sample (Figure S5J). *Within* a given single cell, the abundance of SARS-CoV-2 UMIs ranges from 1 to 12,612, corresponding to 0.01%–98% of all human and viral UMIs per cell.

**Figure S6:**
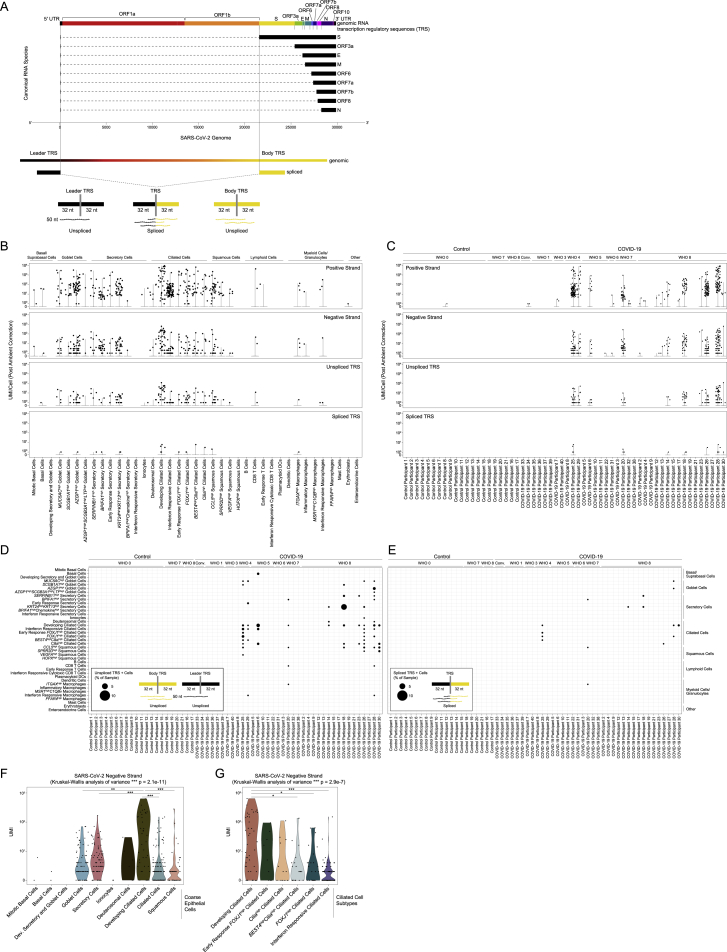
SARS-CoV-2 RNA and cell types containing viral reads, related to Figures 4 and 5 (A) Schematic of method to distinguish unspliced from spliced SARS-CoV-2 RNA species by searching for reads which align across a spliced or genomic Transcription Regulatory Sequence (TRS). (B) Abundance of SARS-CoV-2 aligning UMI/Cell per detailed cell type (following ambient viral RNA correction), split by UMI aligning to the viral positive strand, negative strand, 70-mer region across an unspliced TRS, and 70-mer region across a spliced TRS. (C) Abundance of SARS-CoV-2 aligning UMI/Cell per participant (following ambient viral RNA correction), split by UMI aligning to the viral positive strand, negative strand, 70-mer region across an unspliced TRS, and 70-mer region across a spliced TRS. (D and E) Dot plot of SARS-CoV-2 unspliced TRS aligning UMI (D) and spliced TRS aligning UMI (E) by participant (columns) and detailed cell type (rows). Dot size corresponds to the percent of cells within each sample/cell type containing unspliced (D) or spliced (E) TRS UMI. (F and G) Abundance of SARS-CoV-2 negative strand aligning reads by coarse epithelial cell types (F) and detailed ciliated cell types (G). Statistical significance by Kruskal-Wallis test (p value outside box). Asterisks within box: pairwise Wilcoxon rank sum test, Bonferroni-corrected: ^∗∗∗^p < 0.001, ^∗∗^p < 0.01, ^∗^p < 0.05

To further understand the biological significance of SARS-CoV-2-aligning UMIs within a single cell, and to better identify cells with the highest likelihood of actively supporting viral replication, we analyzed the specific viral sequences and their alignment regions in the viral genome (Figures 4F, 4G, and S6A; Fung and Liu, 2019; Hu et al., 2021; Kim et al., 2020). Single cells containing higher abundances of spliced transcriptional regulatory sequences (TRSs) or negative strand aligning reads are more likely to represent truly virally infected cells with a functional viral replication and transcription complex. We integrate these and other aspects of the host and viral transcriptomes to refine and contextualize our confidence in “SARS-CoV-2 RNA^+^” cells. Critically, the co-detection of host transcriptomic and viral genomic material associated with a single cell barcode cannot definitively establish the presence of intracellular virus and/or productive infection. The majority of SARS-CoV-2-aligning UMIs among SARS-CoV-2 RNA^+^ cells are found heavily biased toward the 3′ end of the genome, attributed to the 3′ UTR, ORF10, and N gene regions, as expected due to poly(A) priming (Figure 4H). A majority (68.7%) of SARS-CoV-2 RNA^+^ cells contain reads aligning to the viral negative strand, increasing the likelihood that many of these cells represent true targets of SARS-CoV-2 virions *in vivo*. In addition to negative strand alignment, we find roughly ∼1/4 of the SARS-CoV-2 RNA^+^ cells contain at least 100 UMIs that map to more than 20 distinct viral genomic locations per cell. When comparing spliced to unspliced UMIs, we find a minor fraction of cells with reads mapping directly across a spliced TRS sequence (4.6%), while 35% of SARS-CoV-2 RNA^+^ cells contain reads mapping across the equivalent 70-mer window around an unspliced TRS.

**Figure 5:**
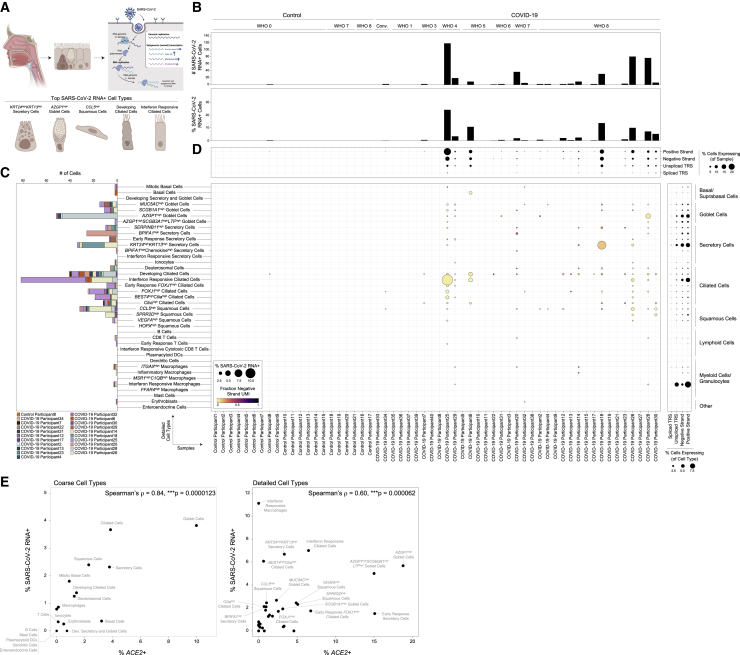
Cellular targets of SARS-CoV-2 in the nasopharynx (A) Summary schematic of top SARS-CoV-2 RNA^+^ cells. (Adapted from “Coronavirus Replication Cycle (Simplified) by BioRender.com (2021). Retrieved from https://app.biorender.com/biorender-templates.) (B) SARS-CoV-2 RNA^+^ cell number (top) and percent (bottom) per participant. (C) Abundance of SARS-CoV-2 RNA^+^ cells by detailed cell type, bars colored by participant. (D) Dot plot of SARS-CoV-2 RNA presence by sample (columns) and detailed cell types (rows). Dot size reflects fraction of a given participant and cell type containing SARS-CoV-2 RNA. Dot color reflects fraction of aligned reads corresponding to the SARS-CoV-2-positive strand (yellow) versus negative strand (black). Top dot plot across columns: alignment of viral reads by participant, separated by RNA species type. Right dot plot across rows: alignment of viral reads by detailed cell type. (E) Percent *ACE2*^+^ cells versus percent SARS-CoV-2 RNA^+^ cells by coarse cell type (left) and detailed cell type (right). See also Figures S5 and S6.

Next, we integrated (1) the strand and splice information among SARS-CoV-2-aligning UMIs, (2) participant-to-participant diversity, and (3) cell type annotations to gain a comprehensive picture of the identity and range of SARS-CoV-2 RNA^+^ cells within the nasopharyngeal mucosa (Figures 5A–5D and S6A–S6E). The majority of SARS-CoV-2 RNA^+^ cells are ciliated, goblet, secretory, or squamous. Highest-confidence SARS-CoV-2 RNA^+^ cells (containing UMIs aligning across a spliced TRS, negative-strand UMIs, and >100 SARS-CoV-2 UMIs/cell) tended to be found among *MUC5AC*^high^ goblet cells, *AZGP1*^high^ goblet cells, *BPIFA1*^high^ secretory cells, *KRT24*^high^*KRT13*^high^ secretory cells, *CCL5*^high^ squamous cells, developing ciliated cells, and each ciliated cell subtype. A high proportion of IFN-responsive macrophages contained SARS-CoV-2 genomic material, and rare *ITGAX*^high^ macrophages are found to contain UMIs aligning to viral negative strand or spliced TRS regions—likely representing myeloid cells that have recently engulfed virally infected epithelial cells or free virions. We did not find major differences in the presumptive cellular tropism by peak COVID-19 severity. The cell types harboring the highest proportions of SARS-CoV-2 RNA^+^ cells represent the same cell types uniquely expanded or induced within COVID-19 participants, such as *KRT24*^high^*KRT13*^high^ secretory cells, *AZGP1*^high^ goblet cells, and IFN-responsive ciliated cells, and contain the highest abundances of *ACE2*-expressing cells (Figure 5E). Developing ciliated cells contain among the highest SARS-CoV-2 RNA molecules per cell, including positive strand, negative strand-aligning reads, and spliced TRS reads (Figure S6F). Among ciliated cell subtypes, IFN-responsive ciliated cells, despite representing one of the most frequent “targets” of viral infection, contain the lowest per-cell abundances of SARS-CoV-2 RNA, potentially reflecting the impact of elevated anti-viral factors curbing high levels of intracellular viral replication (Figure S6G).

### Cell-intrinsic responses to SARS-CoV-2 infection

**Figure 6:**
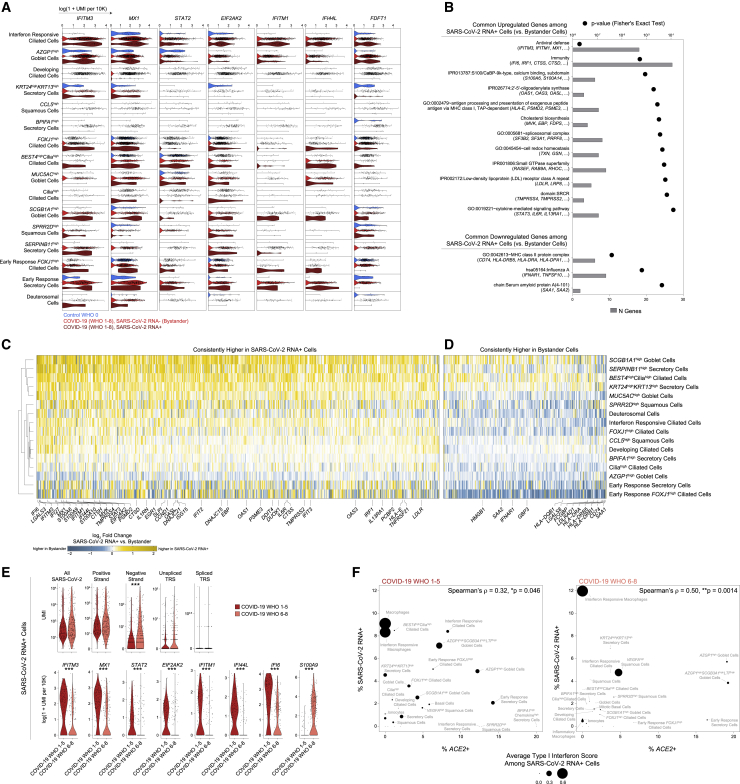
Intrinsic and bystander responses to SARS-CoV-2 infection (A) Violin plots of selected genes upregulated in SARS-CoV-2 RNA^+^ cells in at least three individual cell type comparisons. Blue, control participants; red, bystander cells from COVID-19 participants; dark red, SARS-CoV-2 RNA^+^ cells. (B) Enriched gene ontologies among genes consistently up- or downregulated among SARS-CoV-2 RNA^+^ cells across cell types. (C and D) Heatmap of genes consistently higher in SARS-CoV-2 RNA^+^ cells (C) and higher in bystander cells (D) across multiple cell types. Colors represent log fold changes between SARS-CoV-2 RNA^+^ cells and bystander cells. Yellow, upregulated among SARS-CoV-2 RNA^+^ cells; blue, bystander cells. (E) Top: Violin plots of SARS-CoV-2 aligning reads among SARS-CoV-2 RNA^+^ cells. Statistical significance by Wilcoxon rank sum test. Bottom: select differentially expressed genes between SARS-CoV-2 RNA^+^ cells from participants with mild or moderate COVID-19 (red) versus severe COVID-19 (pink). Statistical significance by likelihood ratio test assuming an underlying negative binomial distribution. ^∗∗∗^ FDR-corrected p < 0.001, ^∗∗^p < 0.01, ^∗^p < 0.05. (F) Percent *ACE2*^+^ cells versus percent SARS-CoV-2 RNA^+^ cells by detailed cell type. Left: cells from participants with mild or moderate COVID-19. Right: cells from participants with severe COVID-19. Point size reflects average type I interferon-specific module score among SARS-CoV-2 RNA^+^ cells. See also Figure S7 and Table S5.

We next mapped both the cell-intrinsic response to direct viral infection as well as the host cell identities that may *potentiate* or *enable* SARS-CoV-2 tropism and replication. To control for variability among different SARS-CoV-2 RNA^+^ cell types and individuals, we compared SARS-CoV-2 RNA^+^ cells to bystander cells of the same cell type and participant (Figures 6A). Many of the genes previously identified as increased within all cells from COVID-19 participants, e.g., anti-viral factors *IFITM3, MX1, IFI44L,* and *IRF1,* are upregulated among SARS-CoV-2 RNA^+^ cells compared to matched bystanders. The majority of genes induced within SARS-CoV-2 RNA^+^ cells are shared *across* diverse cell types, suggesting a conserved anti-viral response and common features that facilitate or restrict infection (Figures 6B–6D, Table S5). SARS-CoV-2 RNA^+^ cells expressed significantly higher abundances of multiple proteases involved in the cleavage of SARS-CoV-2 spike protein, a required step for viral entry (*TMPRSS4, TMPRSS2, CTSS, CTSD*). This suggests that within a given cell type, natural variations in the abundance of genes which support the viral life cycle may partially account for which cells are successfully targeted by the virus.

**Figure S7:**
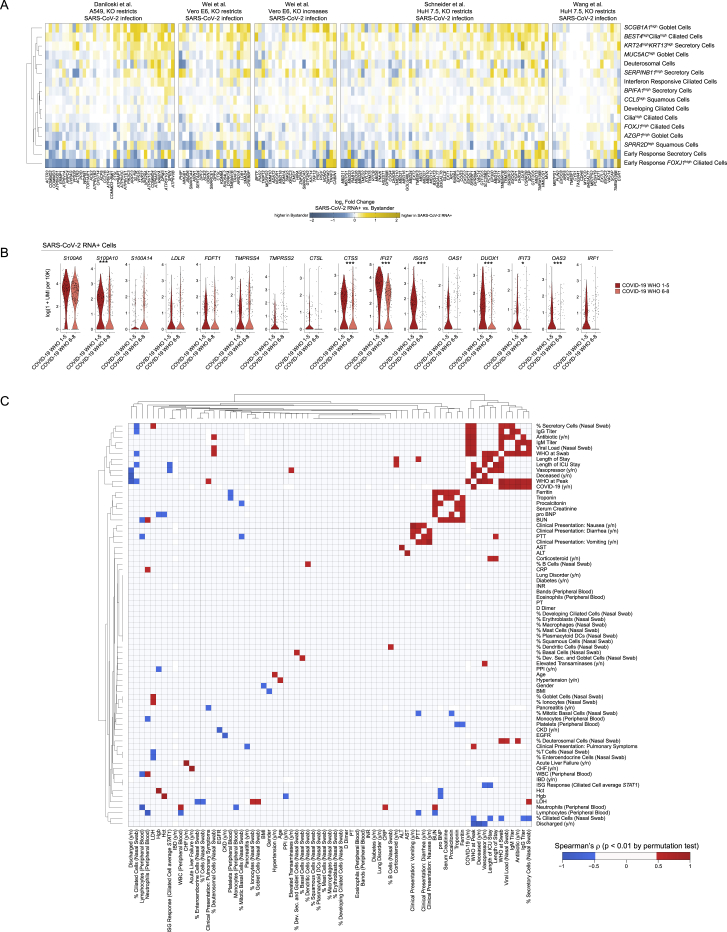
Intrinsic and bystander responses to SARS-CoV-2 infection, related to Figure 6 (A) Heatmaps of log fold changes between SARS-CoV-2 RNA^+^ cells and bystander cells by cell type. Gene sets derived from four CRISPR screens for important host factors in the SARS-CoV-2 viral life cycle. Restricted to cell types with at least 5 SARS-CoV-2 RNA^+^ cells. Yellow: upregulated among SARS-CoV-2 RNA^+^ cells, blue: upregulated among bystander cells. (B) Violin plots of select genes upregulated in SARS-CoV-2 RNA^+^ cells when compared to matched bystanders. Plotting only SARS-CoV-2 RNA^+^ cells from COVID-19 WHO 1-5 participants (red) and COVID-19 WHO 6-8 participants (pink). Statistical significance by likelihood ratio test assuming an underlying negative binomial distribution. ^∗∗∗^ FDR-corrected p < 0.001, ^∗∗^p < 0.01, ^∗^p < 0.05. (C) Heatmap of Spearman’s correlation between 73 clinical parameters, demographic data, or results from scRNA-seq. Includes individuals from healthy (Control WHO 0), COVID-19 mild/moderate (COVID-19 WHO 1-5) and COVID-19 severe (COVID-19 WHO 6-8) groups. Colored squares represent statistically significant associations by permutation test (p < 0.01; red: positive Spearman’s rho; blue: negative Spearman’s rho).

Among the core anti-viral/IFN-responsive gene sets induced within SARS-CoV-2 RNA^+^ cells, we observed repeated and robust upregulation of *IFITM3* and *IFITM1*. Multiple studies have demonstrated that while these two IFN-inducible factors can disrupt viral release from endocytic compartments among a wide diversity of viral species, IFITMs can instead facilitate entry by human betacoronaviruses (Fung and Liu, 2019; Zhao et al., 2014). Therefore, enrichment of these factors within presumptive infected cells may reflect viral hijacking of a conserved host anti-viral responsive pathway. Genes involved in cholesterol and lipid biosynthesis are also upregulated among SARS-CoV-2 RNA^+^ cells, including *FDFT1*, *MVK, FDPS, ACAT2,* and *HMGCS1*, all enzymes involved in the mevalonate synthesis pathway. In addition, SARS-CoV-2 RNA^+^ cells show increased abundance of low-density lipoprotein receptors *LDLR* and *LRP8* compared to matched bystanders. Various genes involved in cholesterol metabolism were recently identified as critical host factors for SARS-CoV-2 replication via CRISPR screens, and additional hits from these datasets are similarly enriched among SARS-CoV-2 RNA^+^ cells in our study (Figure S7A; Daniloski et al., 2021; Schneider et al., 2021; Wang et al., 2021b; Wei et al., 2021). We found increased expression of S100/Calbindin genes such as *S100A6, S100A4,* and *S100A9* among SARS-CoV-2 RNA^+^ cells, which may directly play a role in leukocyte recruitment to infected cells. Finally, we found multiple genes implicated in susceptibility and response to SARS-CoV-2 infection which have not been previously described. *IFNAR1* was substantially increased in many bystander cells compared to both cells from control participants as well as matched SARS-CoV-2 RNA^+^ cells (Figure 6D). Blunting of IFNα signaling via downregulation of *IFNAR1* within SARS-CoV-2 RNA^+^ cells may partially explain high levels of viral replication compared to neighboring cells. Finally, bystander cells expressed significantly higher abundances of MHC-II molecules compared to SARS-CoV-2 RNA^+^ cells, including *HLA-DQB1, HLA-DRB1, HLA-DRB5, HLA-DRA,* and *CD74*.

Anti-viral factors were largely absent from presumptive virally infected cells in participants who developed severe COVID-19, despite equivalent abundances of cell-associated viral UMIs and elevated UMIs/cell aligning to the viral negative strand (Figures 6E and S7B). *EIF2AK2,* which encodes protein kinase R and drives host cell apoptosis following recognition of intracellular double-stranded RNA, is among the most reliably expressed and upregulated genes among SARS-CoV-2 RNA^+^ cells compared to matched bystanders across diverse cell types, suggesting rapid activation of this gene following intrinsic PAMP recognition of SARS-CoV-2 replication intermediates (Krähling et al., 2009). Neither *EIF2AK2* nor IFN-responsive transcription factors such as *STAT1* and *STAT2* were expressed within SARS-CoV-2 RNA^+^ cells from participants who developed severe COVID-19 (Figure 6E). This suggests that direct sensing of intracellular viral products may amplify IFN-responsive and anti-viral gene upregulation, though these pathways are only induced among SARS-CoV-2 RNA^+^ cells from participants with mild or moderate COVID-19 (Figure 6F). Together, this suggests a failure of the *intrinsic* immune response to viral infection among nasal epithelial cells in individuals who develop severe COVID-19.

## Discussion

We present a comprehensive map of SARS-CoV-2 infection of the human nasopharynx using scRNA-seq. We hypothesize that the host response at the site of initial infection, the nasal mucosa, is an essential determinant of overall COVID-19 disease trajectory. By dissecting the nature of host-pathogen interactions at this primary viral target across the spectrum of disease trajectories, we characterize both protective and pathogenic responses to SARS-CoV-2 infection. This enables us to begin to untangle the myriad factors that may restrict viral infection to the upper respiratory tract or support the development of severe lower respiratory tract disease (Figure S7C). Our study defines major compositional differences in the nasal epithelia during COVID-19 and directly relates these to NP viral load, cellular tropism, and cell-intrinsic responses to SARS-CoV-2. Further, we identify marked variability in the induction of anti-viral gene expression that is associated with peak disease severity and may *precede* development of severe respiratory damage. We find that anti-viral gene expression is profoundly blunted in cells isolated from individuals who develop severe disease, even in cells containing SARS-CoV-2 RNA.

Individuals who develop severe COVID-19 have equivalent or even elevated levels of nasal SARS-CoV-2 RNA at the time of sampling and contained expanded inflammatory and type II-IFN-responsive macrophages compared to mild or moderate cases. Indeed, published peripheral immune studies comparing mild and severe COVID-19 also observe diminished type I and type III IFN abundances in severe cases and note restricted IFN-stimulated gene expression among circulating immune cells (Galani et al., 2021; Hadjadj et al., 2020; Stephenson et al., 2021). Other human betacoronaviruses (MERS and SARS-CoV) exhibit multiple strategies to avoid triggering pattern recognition receptor pathways, including degradation of host mRNA within infected cells (Kamitani et al., 2009; Lokugamage et al., 2015), sequestration of viral replication intermediates (e.g., double stranded RNA) from host sensors (Knoops et al., 2008), and direct inhibition of immune effector molecules (Fung and Liu, 2019; Krähling et al., 2009; Menachery et al., 2014), leading to diminished induction of anti-viral pathways and blunted autocrine and paracrine IFN signaling. Strategies to avoid innate immune recognition have now been extended to SARS-CoV-2 as well, indicating that avoiding host recognition is likely an essential aspect of viral success (Banerjee et al., 2020; Konno et al., 2020; Snijder et al., 2020). The close association we observe between peak disease severity and weak anti-viral gene expression among nasal epithelial cells is intriguing given recent observations of inborn defects in TLR3, IRF7, IRF9, and IFNAR1, or antibody-mediated neutralization of type I IFN responses within individuals who develop severe COVID-19 (Bastard et al., 2020, 2021; Combes et al., 2021; Wang et al., 2021a; Zhang et al., 2020). Taken together, these findings suggest that severe infection arises in the setting of impaired intrinsic epithelial anti-viral immunity, even in the absence of detectable type I IFN-targeting autoantibodies. We surmise that the combined effects of a viral strain with naturally poor IFN induction and defects in immune or intrinsic epithelial anti-viral responses within the nasal mucosa may predispose to severe disease via enhanced viral replication in the upper airway, eventually leading to immunopathology characteristic of severe COVID-19.

Among individuals who develop severe COVID-19 in our cohort, we observe unique recruitment of highly inflammatory macrophages that represent the major tissue sources of proinflammatory cytokines including *IL1B*, *TNF*, *CXCL8*, *CCL2*, *CCL3*, and *CXCL9/10/11*—of likely relation to the immune dysregulation characterized by elevation of the same factors in the periphery in severe disease and observed in lung tissue among those who succumbed to COVID-19 (Delorey et al., 2021; Lucas et al., 2020). In addition, we note specific upregulation of alarmins *S100A8/S100A9* (i.e., calprotectin) among epithelial cells in the severe COVID-19 group compared to mild or moderate and control counterparts, and even higher expression of *S100A9* within SARS-CoV-2 RNA^+^ cells from those same individuals. A recent study identified these as potential biomarkers of severe COVID-19 and proposed that these factors directly drive excessive inflammation and precede the massive cytokine release characteristic of late disease (Silvin et al., 2020). Our work suggests that severe COVID-19-specific expression of calprotectin may originate within the virally infected nasal epithelia. Further work to understand the epithelial cell regulation of *S100A8/A9* gene expression may help clarify maladaptive responses to SARS-CoV-2 infection.

Finally, we provide a direct investigation into the host factors that enable or restrict SARS-CoV-2 replication within epithelial cells *in vivo*. We recapitulate expected “hits” based on well-described host factors involved in viral replication—e.g., *TMPRSS2* and *TMPRSS4* enrichment among presumptive virally infected cells. In accordance with previous studies into the nasal epithelial response to influenza infection (Cao et al., 2020), we observe bystander epithelial cell upregulation of both MHC-I and MHC-II family genes; however, we find that SARS-CoV-2 RNA^+^ cells only express MHC-I, and poorly express MHC-II genes compared to matched bystanders. To our knowledge, downregulation of host cell pathways for antigen presentation by coronaviruses has not been previously described. A recent study found that CIITA and CD74 can intrinsically block entry of a range of viruses (including SARS-CoV-2) via endosomal sequestration, and therefore cells that upregulate these (and other) components of MHC-II machinery may naturally restrict viral entry (Bruchez et al., 2020).

Together, our work and that of our colleagues suggest that several mechanisms that restrict interferon-mediated viral control in the upper respiratory epithelium can enable progression to severe COVID-19, that these causes may be multifactorial and rooted in human diversity, and yet they converge on impaired intrinsic immunity to SARS-CoV-2 in nasal epithelial cells. Further, it suggests that there may be a clinical window in which severe disease can be subverted by focusing preventative or therapeutic interventions early within the nasopharynx (Feld et al., 2020; Hoagland et al., 2021; Monk et al., 2021; Wang et al., 2020a), bolstering anti-viral responses and curbing pathological inflammatory signaling prior to development of severe respiratory dysfunction or systemic disease.

### Limitations of the study

A major difficulty in understanding SARS-CoV-2 infection *in vivo* at early, disease-relevant time points lies in limited strategies for sampling the upper airway cellular makeup. We collected viable cells using NP swabs—a minimally invasive clinical procedure that we were able to apply in both an ambulatory setting and the intensive care unit at the height of the summer 2020 COVID-19 surge in Mississippi. The simplicity of this procedure, in comparison to BAL or biopsy-based measurements, represents a major advantage. However, our pipeline, as implemented, has key limitations arising from viably freezing the NP swabs (e.g., cell loss, limited cell recovery). We have confirmed that freezing cells directly on a NP swab does not induce major differences in the recovered cell composition of the nasal mucosa among healthy donors, but we cannot exclude alterations specific to individuals with COVID-19. Consequently, with limited cellular capture per swab, we potentially under-sample features of the nasal epithelium, which impacts our ability to detect and compare low-abundance cell subtypes—such as rare immune cells with <0.5% frequency in the nasal epithelium (e.g., mast cells, plasmacytoid DCs). Further, though we are able to recover SARS-CoV-2 aligning reads in more than 80% of COVID-19 participants, we recovered few cells *per participant* that can be assigned as “SARS-CoV-2 RNA” with high confidence. Accordingly, we detect remarkably high heterogeneity in SARS-CoV-2-targeted cell subtypes across individuals, which may be due to incomplete recovery of the full distribution of SARS-CoV-2-targeted cells in any single donor. Finally, our data represent the population served by the University of Mississippi Medical Center, and further work will be required to understand how these data relate to the general population. Multiple cohorts replicated by distinct research groups across clinical centers and patient populations will be critical to understand the generalizability of our findings.

## STAR★Methods

### Key resources table

**Table:** 

REAGENT or RESOURCE	SOURCE	IDENTIFIER
**Antibodies**		

PerCP-Cy5.5-conjugated anti-human CD45 (clone: HI30)	BioLegend	Cat# 304027; RRID: AB_1236444
Brilliant Violet 711-conjugated anti-human CD3 (clone: SK7)	BioLegend	Cat# 344837; RRID: AB_2565826
APC-Cy7-conjugated anti-human CD8 (clone: SK1)	BioLegend	Cat# 344714; RRID: AB_2044006
PE-conjugated anti-human CD4 (clone: RPA-T4)	BioLegend	Cat# 300508; RRID: AB_314076
Brilliant Violet 786-conjugated anti-human CD326 (clone: 9C4)	BioLegend	Cat# 324237; RRID: AB_2632936
PE-Cy5-conjugated anti-human CD19 (clone: HIB19)	BioLegend	Cat# 302209; RRID: AB_314239
PE-Cy7-conjugated anti-human CD66b (clone: G10F5)	BioLegend	Cat# 305115; RRID: AB_2566604
Brilliant Violet 650-conjugated anti-human CD11c (clone: Bu15)	BioLegend	Cat# 337237; RRID: AB_2721551
FITC-conjugated anti-human CD14 (clone: M5E2)	BioLegend	Cat# 301804; RRID: AB_314186
Brilliant Violet 421-conjugated anti-human CD56 (clone: 5.1H11)	BioLegend	Cat# 362551; RRID: AB_2566060
PE-Cy7-conjugated anti-human CD49f (clone: GoH3)	Biolegend	Cat# 313621; RRID: AB_2561704
PE-conjugated anti-human CD66c (clone: B6.2/CD66)	BD	Cat# 551478; RRID: AB_394216
APC-Cy7-conjugated anti-human CD271 (clone: ME20.4)	Biolegend	Cat# 345125; RRID: AB_2876654
FITC-conjugated Tubulin-alpha (clone: 10D8)	Biolegend	Cat# 627905; RRID: AB_893643

**Biological Samples**		

Human nasopharyngeal swabs	University of Mississippi Medical Center	IRB#2020-0065
Human plasma	University of Mississippi Medical Center	IRB#2020-0065

**Chemicals, Peptides, and Recombinant Proteins**		

2-Mercaptoethanol	Sigma	Cat# M3148-25ML
RLT Buffer	QIAGEN	Cat# 79216
dNTP	New England BioLabs	Cat# N0447L
RNase Inhibitor	Fisher Scientific	Cat# AM2696
Maxima RNaseH-minus RT Enzyme	Fisher Scientific	Cat# EP0753
AMPure RNAClean XP RNA-SPRI beads	Beckman Coulter	Cat# A63987
AMPure XP SPRI beads	Beckman Coulter	Cat# A63881
Guanidinium thiocyanate	Sigma	Cat# AM9422
Sarkosyl	Sigma	Cat# L7414
Exonuclease I	New England BioLabs	Cat# M0293S
Klenow Fragment	New England BioLabs	Cat #M0212L
Accutase	Sigma	Cat# A6964
Dithiothreitol (DTT)	Sigma	Cat# 43816

**Critical Commercial Assays**		

Nextera XT DNA Library Preparation Kit	Illumina	Cat# FC-131-1096
NextSeq 500/550 High Output v2 (75 cycles)	Illumina	Cat# FC-404-2005
Kapa HiFi HotStart ReadyMix	Kapa Biosystems	Cat# KK2602
MACOSKO-2011-10 mRNA Capture Beads	ChemGenes	Cat# NC0927472
High Sensitivity D5000 ScreenTape	Agilent	Cat# 5067-5592
Qubit dsDNA High-Sensitivity kit	ThermoFisher	Cat# Q32854
AbC Total Antibody Compensation Beads	Life Technologies	Cat# A10497
ArC Amine Reactive Compensation Beads	Life Technologies	Cat# A10346
Human TruStain FcX™ (Fc receptor blocking solution)	BioLegend	Cat# 422302
BD Fixation Buffer	BD Biosciences	Cat# 554655
Fixable Aqua Dead Cell Stain	ThermoFisher	Cat# L34965
OmicsArray Antigen Microarrays	GeneCopoeia	Cat# PA012

**Deposited Data**		

Processed and raw data, scRNA-seq of all cells from nasopharyngeal swabs	This paper	Single Cell Portal: https://singlecell.broadinstitute.org/single_cell/study/SCP1289
Custom SARS-CoV-2 FASTA and GTF	This paper	Github: https://github.com/ShalekLab/SARSCoV2-genome-reference
Human reference genome NCBI build 38 (GRCh38)	Genome Reference Consortium	GEO: https://www.ncbi.nlm.nih.gov/projects/genome/assembly/grc/human/
SARS-CoV-2 Genome Reference	Kim et al., 2020	Github: https://github.com/hyeshik/sars-cov-2-transcriptome
Population RNA-seq data, human basal cells	Ziegler et al., 2020	Single Cell Portal: https://singlecell.broadinstitute.org/single_cell/study/SCP822

**Oligonucleotides**		

Seq-Well ISPCR: AAG CAG TGG TAT CAA CGC AGA GT	Integrated DNA Technologies	N/A
Custom Read 1 Primer: GCC TGT CCG CGG AAG CAG TGG TAT CAA CGC AGA GTA C	Integrated DNA Technologies	N/A
Seq-Well 5' TSO: AAG CAG TGG TAT CAA CGC AGA GTG AAT rGrGrG	Integrated DNA Technologies	N/A
Seq-Well Custom P5-SMART PCR hybrid oligo: AAT GAT ACG GCG ACC ACC GAG ATC TAC ACG CCT GTC CGC GGA AGC AGT GGT ATC AAC GCA GAG TAC	Integrated DNA Technologies	N/A
Seq-Well dN-SMRT oligo: AAG CAG TGG TAT CAA CGC AGA GTG ANN NGG NNN B	Integrated DNA Technologies	N/A

**Software and Algorithms**		

R Project for Statistical Computing 4.0.2	R Core Team	https://www.r-project.org
R package – Seurat v3.2.2	Github	https://github.com/satijalab/seurat
R package – DESeq2 v1.30.0	Bioconductor	https://bioconductor.org/packages/DESeq2/
R package – Circlize v0.4.11	CRAN	https://CRAN.R-project.org/package=circlize
R package – ggplot2 v3.3.2	CRAN	https://CRAN.R-project.org/package=ggplot2
R package – ComplexHeatmap v2.7.3	Bioconductor	https://bioconductor.org/packages/ComplexHeatmap/
R package – fgsea v1.16.0	Bioconductor	https://bioconductor.org/packages/fgsea/
Python Programming Language v3.8.3	Python	https://www.python.org
Python package scVelo v0.3.0	Bergen et al., 2020	https://scvelo.readthedocs.io/
CellBender	Fleming et al., 2019	https://cellbender.readthedocs.io/
Cumulus	Li et al., 2020	https://cumulus.readthedocs.io/
Prism 6	GraphPad Software	https://www.graphpad.com/scientific-software/prism/
STAR	Github	https://github.com/alexdobin/STAR
FlowJo v10.7.1	TreeStar Inc.	N/A

### Resource availability

#### Lead Contact

Further information and requests for resources and reagents should be directed to and will be fulfilled by the lead contact, Dr. Jose Ordovas-Montanes (jose.ordovas-montanes@childrens.harvard.edu).

#### Materials Availability

This study did not generate new unique reagents.

### Experimental model and subject details

Eligible participants were recruited from outpatient clinics, medical surgical units, intensive care units (ICU), or endoscopy units at the University of Mississippi Medical Center (UMMC) between April 2020 and September 2020. The UMMC Institutional Review Board approved the study under IRB#2020-0065. All participants or their legally authorized representative provided written informed consent. Participants were eligible for inclusion in the COVID-19 group if they were at least 18 years old, had a positive nasopharyngeal swab for SARS-CoV-2 by PCR, had COVID-19 related symptoms including fever, chills, cough, shortness of breath, and sore throat, and weighed more than 110 lb. Participants were eligible for the Control group if they were at least 18 years old, had a current negative SARS-CoV-2 test (PCR or rapid antigen test), and weighed more than 110 lb. Participants were considered “Convalescent” if they met the criteria of the Control group, however had previously tested SARS-CoV-2 PCR positive and diagnosed with COVID-19, and their symptoms had subsided for at least 40 days. Convalescent samples were treated as an independent group, and excluded from comparisons between “Control” and “COVID-19” groups. Exclusion criteria for the cohort included a history of blood transfusion within 4 weeks and subjects who could not be assigned a definitive COVID-19 diagnosis from nucleic acid testing. 35 individuals with COVID-19 were included, both male (n = 19) and female (n = 16). For the Control group, 21 participants were included – 11 identified as male, 10 as female. The median age of COVID-19 participants was 55 years old; the median age of Control participants was 62 years old. Among hospitalized COVID-19 participants, the median day NP swabs were collected was hospital day 2 (inter-quartile range: 1, range 1-9). COVID-19 participants were classified according to the 8-level ordinal scale proposed by the WHO representing their peak clinical severity and level of respiratory support required (World Health Organization, 2020) (Table 1, Figures S1A–S1E). Notably, for many participants the peak severity differed from their clinical severity score on the day of nasopharyngeal swab (Figures S1F, S1G). We confirmed that basic health and demographic information including: age, sex, BMI, race/ethnicity, and presence of co-morbidities was balanced across disease groups (assessed using Chi-square test for categorial variables and Kruskal-Wallis test for continuous metrics, all comparisons non-significant, p > 0.05, Figures S1C–S1E, S7C, Table 1). We additionally performed a correlation analysis among each of our clinical features to understand the relationships among distinct clinical pictures (e.g., severity score), laboratory findings (e.g., CRP), and demographic information (e.g., age), and how these relate to our major findings (e.g., secretory cell proportion, viral load, ISG induction, Figure S7C).

### Method details

#### Sample Collection and Biobanking

Nasopharyngeal samples were collected by a trained healthcare provider using FLOQSwabs (Copan flocked swabs) following the manufacturer’s instructions. Collectors would don personal protective equipment (PPE), including a gown, non-sterile gloves, a protective N95 mask, a bouffant, and a face shield. The patient’s head was tilted back slightly, and the swab inserted along the nasal septum, above the floor of the nasal passage to the nasopharynx until slight resistance was felt. The swab was then left in place for several seconds to absorb secretions and slowly removed while rotating swab. The swab was then placed into a cryogenic vial with 900 μL of heat inactivated fetal bovine serum (FBS) and 100 μL of dimethyl sulfoxide (DMSO). Vials were placed into a Mr. Frosty Freezing Container (Thermo Fisher Scientific) for optimal cell preservation. A Mr. Frosty containing the vials was placed in a cooler with dry ice for transportation from patient areas to the laboratory for processing. Once in the laboratory, the Mr. Frosty was placed into a −80°C freezer overnight, and on the next day, the vials were moved to liquid nitrogen storage containers.

#### Dissociation and Collection of Viable Single Cells from Nasopharyngeal Swabs

Swabs in freezing media (90% FBS/10% DMSO) were stored in liquid nitrogen until immediately prior to dissociation. A detailed sample protocol can be found here: https://protocols.io/view/human-nasopharyngeal-swab-processing-for-viable-si-bjhkkj4w.html. (Tang et al., 2020). This approach (Figure S1I) ensures that all cells and cellular material from the nasal swab (whether directly attached to the nasal swab, or released during the washing and digestion process), are exposed first to DTT for 15 min, followed by an Accutase digestion for 30 min. Briefly, nasal swabs in freezing media were thawed, and each swab was rinsed in RPMI before incubation in 1 mL RPMI/10 mM DTT (Sigma) for 15 min at 37°C with agitation. Next, the nasal swab was incubated in 1 mL Accutase (Sigma) for 30 min at 37°C with agitation. The 1 mL RPMI/10 mM DTT from the nasal swab incubation was centrifuged at 400 g for 5 min at 4°C to pellet cells, the supernatant was discarded, and the cell pellet was resuspended in 1 mL Accutase and incubated for 30 min at 37°C with agitation. The original cryovial containing the freezing media and the original swab washings were combined and centrifuged at 400 g for 5 min at 4°C. The cell pellet was then resuspended in RPMI/10 mM DTT, and incubated for 15 min at 37°C with agitation, centrifuged as above, the supernatant was aspirated, and the cell pellet was resuspended in 1 mL Accutase, and incubated for 30 min at 37°C with agitation. All cells were combined following Accutase digestion and filtered using a 70 μm nylon strainer. The filter and swab were washed with RPMI/10% FBS/4 mM EDTA, and all washings combined. Dissociated, filtered cells were centrifuged at 400 g for 10 min at 4°C, and resuspended in 200 μL RPMI/10% FBS for counting. Cells were diluted to 20,000 cells in 200 μL for scRNA-seq. For the majority of swabs, fewer than 20,000 cells total were recovered. In these instances, all cells were input into scRNA-seq.

We directly tested whether cell types collected from NP swabs following cryopreservation were representative of the cellular composition extracted from a freshly-swabbed nasopharyngeal epithelium, or if certain cell types were lost during freezing (Figure S2B-S2G). Recovery of viable cells, technical metrics of single-cell library quality, and cellular proportions after clustering and analysis were all largely stable between matched fresh and cryopreserved swabs taken from the same individual. Importantly, no “new” cell types (from healthy participants) were recovered from the freshly processed samples.

#### Flow Cytometry of Cells Isolated from Nasopharyngeal Swabs

Single-cell suspensions were isolated from nasopharyngeal swabs of healthy donors, as described above. Cells were first stained with Fixable Aqua Dead Cell Stain (Thermo Fisher Scientific) for 15 min to assess viability. Cells were washed with staining buffer (PBS/2% FBS), and then treated with Human TruStain FcX (Fc receptor blocking solution, Cat. No. 422302, BioLegend) for 5 min. For quantification of immune cell subtypes: cells were stained with a surface marker antibody cocktail on ice for 15 min, which contained PerCP-Cy5.5-conjugated anti-human CD45 (clone: HI30, BioLegend), Brilliant Violet 711-conjugated anti-human CD3 (clone: SK7, BioLegend), APC-Cy7-conjugated anti-human CD8 (clone: SK1, BioLegend), PE-conjugated anti-human CD4 (clone: RPA-T4, BioLegend), Brilliant Violet 786-conjugated anti-human CD326 (clone: 9C4, BioLegend), PE-Cy5-conjugated anti-human CD19 (clone: HIB19, BioLegend), PE-Cy7-conjugated anti-human CD66b (clone: G10F5, BioLegend), Brilliant Violet 650-conjugated anti-human CD11c (clone: Bu15, BioLegend), FITC-conjugated anti-human CD14 (clone: M5E2, BioLegend), and Brilliant Violet 421-conjugated anti-human CD56 (clone: 5.1H11, BioLegend). For quantification of epithelial subsets: cells were stained with an antibody cocktail containing PerCP-Cy5.5-conjugated anti-human CD45 (clone: HI30, BioLegend), PE-Cy7-conjugated anti-human CD49f (clone: GoH3, BioLegend), PE-conjugated anti-human CD66c (clone: B6.2/CD66, BD), APC-Cy6-conjugated anti-human CD271 (clone: ME20.4, Biolegend), and FITC-conjugated tubulin alpha (clone: 10D8, Biolegend) (Bonser et al., 2021). Finally, cells were fixed using BD Fixation Buffer (Cat. No. 554655, BD Biosciences). AbC Total Antibody Compensation Beads (Cat. No. A10497, Life Technologies) and ArC Amine Reactive Compensation Beads (Cat. No. A10346, Life Technologies) were used for compensation. Data were acquired on an LSRFortessa flow cytometer (BD Biosciences) using BD FACSDiva software, and analyzed by FlowJo software (Version 10.7.1, Tree Star Inc.).

#### Detection of Autoantibodies Binding Human Interferons

Plasma from a subset of 25 participants (5 healthy controls, 12 mild/moderate COVID-19, 8 severe COVID-19) was collected on the same day as nasopharyngeal swab. Autoantibodies were assessed using a commercial microarray-based platform (GeneCopoeia). Briefly, participant plasma was hybridized to distinct microarray spots containing 120 native human and viral antigens spotted onto nitrocellulose fibers (adhered to glass slides). Next, the slides were incubated with fluorescently-coupled anti-IgG or anti-IgM secondary antibodies, and microarrays were scanned using a GenePix 4400A microarray scanner. Raw fluorescence data was normalized to PBS controls on each slide.

#### Single Cell RNA-Sequencing

Seq-Well S^3^ was run as previously described (Aicher et al., 2019; Gierahn et al., 2017; Hughes et al., 2019). Briefly, a maximum of 20,000 single cells were deposited onto Seq-Well arrays preloaded with a single barcoded mRNA capture bead (ChemGenes) per well (Macosko et al., 2015). cells were allowed to settle by gravity into wells for 10 min, after which the arrays were washed with PBS and RPMI, and sealed with a semi-permeable membrane for 30 min, and incubated in lysis buffer (5 M guanidinium thiocyanate/1 mM EDTA/1% BME/0.5% sarkosyl) for 20 min. Arrays were then incubated in a hybridization buffer (2M NaCl/8% v/v PEG8000) for 40 min, and then the beads were removed from the arrays and collected in 1.5 mL tubes in wash buffer (2M NaCl/3 mM MgCl_2_/20 mM Tris-HCl/8% v/v PEG8000). Beads were resuspended in a reverse transcription master mix, and reverse transcription, exonuclease digestion, second-strand synthesis, and whole transcriptome amplification were carried out as previously described. Libraries were generated using Illumina Nextera XT Library Prep Kits and sequenced on NextSeq 500/550 High Output 75 cycle v2.5 kits to an average depth of 180 million aligned reads per array: read 1: 21 (cell barcode, UMI), read 2: 50 (digital gene expression), index 1: 8 (N700 barcode).

### Quantification and statistical analysis

#### Data Preprocessing and Quality Control

Pooled libraries were demultiplexed using bcl2fastq (v2.17.1.14) with default settings (mask_short_adapter_reads 10, minimum_trimmed_read_length 10, implemented using Cumulus, snapshot 4, https://cumulus.readthedocs.io/en/stable/bcl2fastq.html) (Li et al., 2020). Libraries were aligned using STAR within the Drop-Seq Computational Protocol (https://github.com/broadinstitute/Drop-seq) and implemented on Cumulus (https://cumulus.readthedocs.io/en/latest/drop_seq.html, snapshot 9, default parameters) (Macosko et al., 2015). A custom reference was created by combining human GRCh38 (from CellRanger version 3.0.0, Ensembl 93) and SARS-CoV-2 RNA genomes. The SARS-CoV-2 viral sequence and GTF are as described in Kim et al., 2020 (https://github.com/hyeshik/sars-cov-2-transcriptome, BetaCov/South Korea/KCDC03/2020 based on NC_045512.2) (Kim et al., 2020). The GTF includes all CDS regions (as of this annotation of the transcriptome, the CDS regions completely cover the RNA genome without overlapping segments), and regions were added to describe the 5′ UTR (“SARSCoV2_5prime”), the 3′ UTR (“SARSCoV2_3prime”), and reads aligning to anywhere within the Negative Strand (“SARSCoV2_NegStrand”). Trailing A’s at the 3′ end of the virus were excluded from the SARS-CoV-2 FASTA, as these were found to drive spurious viral alignment in pre-COVID19 samples. Finally, additional small sequences were appended to the FASTA and GTF that differentiate reads that align to the 70-nucleotide region around the viral TRS sequence – either across the intact, unspliced genomic sequences (e.g., named “SARSCoV2_Unspliced_S” or “SARSCoV2_Unspliced_Leader”) or various spliced RNA species (e.g., “SARSCoV2_Spliced_Leader_TRS_S”), see schematics in Figures 4F, 4G, Figures S6A. Alignment references were tested against a diverse set of pre-COVID-19 samples and *in vitro* SARS-CoV-2 infected human bronchial epithelial cultures (Ravindra et al., 2020) to confirm specificity of viral aligning reads. Aligned cell-by-gene matrices were merged across all study participants, and cells were filtered to eliminate barcodes with fewer than 200 UMI, 150 unique genes, and greater than 50% mitochondrial reads. Swabs from 58 individuals are included in the study. Three additional swabs were thawed and processed but contained no high-quality cell barcodes after sequencing (**NB**: these samples contained < 5,000 viable cells prior to Seq-Well array loading).This resulted in a final dataset of 32,871 genes and 32,588 cells across 58 study participants (35 SARS-CoV-2+, 23 SARS-CoV-2-). Preprocessing, alignment, and data filtering was applied equivalently to samples from the fresh versus frozen cohort. For analysis of RNA velocity, we also recovered both exonic and intronic alignment information using DropEst (Cumulus (https://cumulus.readthedocs.io/en/latest/drop_seq.html, snapshot 9, dropest_velocyto true, run_dropest true) (Petukhov et al., 2018).

#### Cell Clustering and Annotation

Dimensionality reduction, cell clustering, and differential gene analysis were all achieved using the Seurat (v3.1.5) package in R programming language (v3.0.2) (Stuart et al., 2019). Dimensionality reduction was carried out by running principal components analysis (PCA) over the 3,483 most variable genes with dispersion > 0.8 (tested over a range of dispersion > 0.7 to dispersion > 1.2; dispersion > 0.8 was determined as optimal based on number of variable genes, and general stability of clustering results across these cutoffs was confirmed). Only variable genes from human transcripts were considered for dimensionality reduction and clustering. Using the Jackstraw function within Seurat, we selected the first 36 principal components that described the majority of variance within the dataset, and used these for defining a nearest neighbor graph and Uniform Manifold Approximation and Projection (UMAP) plot. Cells were clustered using Louvain clustering, and the resolution parameter was chosen by maximizing the average silhouette score across all clusters. Differentially expressed genes between each cluster and all other cells were calculated using a likelihood ratio test, implemented with Seurat’s FindAllMarkers function, test.use set to “bimod” (McDavid et al., 2013). Clusters were merged if they failed to contain > 25 significantly differentially expressed genes (FDR < 0.001). We proceeded iteratively through each cluster and subcluster until “terminal” cell subsets/cell states were identified – we defined “terminal” cell states when PCA and Louvain clustering did not confidently identify additional sub-states, as measured by abundance of differentially expressed genes between potential clusters (often > 25 cluster-specific marker genes with FDR < 0.001). Among two samples, we recovered erythroblast-like cells, defined by expression of hemoglobin subunits including *HBB and HBA2* (these were from swabs noted to be slightly red-tinged on day of processing). For visualization in Figure 2, we pooled all cells determined to be of epithelial origin from coarse-grained annotation (ciliated cells, secretory cells, goblet cells, basal cells, mitotic basal cells, developing secretory and goblet cells, developing ciliated cells, squamous cells, deuterosomal cells, and ionocytes) and used the methods for dimensionality reduction as above (dispersion cutoff > 1, 30 principal components). We applied similar approaches for immune cell types (Figure S3), including iterative subclustering to resolve and annotate all constituent cells types and subtypes. Gene module scores were calculated using the AddModuleScore function within Seurat.

Seven major epithelial types were recovered from this dataset, annotated according to prior knowledge of the composition and developmental relationships between cells of the upper airway and nasal mucosa (Deprez et al., 2020; Rao Tata and Rajagopal, 2017). Basal cells represent the main stem/progenitor cells of the nasal epithelium, and are classically defined by *TP63*, *KRT5*, and *KRT15* expression (Rock et al., 2009). Goblet cells generate the contents of mucus via secretion of mucin proteins (e.g., *MUC5AC, MUC5B*), which play a critical role in lubricating the respiratory epithelium and trapping inhaled particles and pathogens. Secretory cells are a distinct exocrine cell of the nasal epithelium, and are distinguished from goblet cells by the absence of mucin production and preferential expression of antimicrobial molecules (e.g., *BPIFA1)* (Bingle and Craven, 2002). We designated a small population of cells “developing secretory and goblet cells” based on their lower expression of classic secretory/goblet cell genes, as well as persistent expression of some basal cell markers (e.g., persistent *COL7A1* and *DST* expression, but diminishing *KRT5*, *KRT15* expression). A specialized subtype of secretory/club cell, ionocytes, were recently discovered in lung tissue, and regulate mucus viscosity and are the major expressors of *CFTR* (mutations in the CFTR gene underlie the disease cystic fibrosis) (Montoro et al., 2018; Plasschaert et al., 2018). Ciliated cells are the most numerous cell type of the upper respiratory epithelium, whose hair-like cilia move mucus and debris out of the respiratory tract. Deuterosomal cells are another recently discovered respiratory cell type, and are hypothesized to represent an intermediate cell state as secretory/goblet cells trans-differentiate into ciliated cells. Deuterosomes are cellular structures that produce the numerous centrioles required to nucleate multi-ciliated structures on ciliated cells; the genes to produce deuterosomes are upregulated within deuterosomal cells, such as *DEUP1* and *CCNO* (Garcıá et al., 2019; Revinski et al., 2018). Finally, squamous cells of the nasopharyngeal mucosa are flat cells that form barrier structures of the oropharyngeal and anterior nasal mucosa.

Among some cell types, we did not find additional within-type diversity, and thus the “coarse” annotations (Figure 2A) are equivalent to the “detailed” identities (Figure 2D). We annotated epithelial subtypes according to the following groups and representative markers: goblet cells were split into 4 distinct sets: *MUC5AC*^high^ goblet cells, which lacked additional specialized markers beyond classic goblet cell identifiers, *SCGB1A1*^high^ goblet cells, *AZGP1*^high^ goblet cells, and *AZGP1*^high^*SCGB3A1*^high^*LTF*^high^ goblet cells. Secretory cells were divided into 6 distinct detailed subtypes: *SERPINB11*^high^ secretory cells (which, similar to *MUC5AC*^high^ goblet cells, represented a more “generic” or un-differentiated secretory cell phenotype), *BPIFA1*^high^ secretory cells, early response secretory cells (which expressed genes such as *JUN, EGR1, FOS, NR4A1*), *KRT24*^high^*KRT13*^high^ secretory cells (which are similar to previously-described KRT13+ “hillock” cells (Deprez et al., 2020; Montoro et al., 2018)), *BPIFA1*^high^chemokine^high^ secretory cells (chemokines include *CXCL8, CXCL2, CXCL1,* and *CXCL3),* and interferon responsive secretory cells (defined by higher expression of broad anti-viral genes including *IFITM3, IFI6,* and *MX1*). Subsets of squamous cells were also found – detailed squamous cell subtypes include *CCL5*^high^ squamous cells, *VEGFA*^high^ squamous cells (which express multiple vascular endothelial genes including *VEGFA and VWF*), *SPRR2D*^high^ squamous cells (which, in addition to *SPRR2D*, express the highest abundances of multiple SPRR- genes including *SPRR2A, SPRR1B, SPRR2E,* and *SPRR3*), and *HOPX*^high^ squamous cells. Finally, ciliated cells could be further divided into 5 distinct subtypes: interferon responsive ciliated cells (expressing anti-viral genes similar to other “interferon responsive” subsets, such as *IFIT1, IFIT3, IFI6*), *FOXJ1*^high^ ciliated cells, early response *FOXJ1*^high^ ciliated cells (which, in addition to high *FOXJ1*, also express higher abundances of genes such as *JUN*, *EGR1*, *FOS* than other ciliated cell subtypes), cilia^high^ ciliated cells (which broadly express the highest abundances of structural cilia genes, such as *DLEC1* and *CFAP100),* and *BEST4*^high^cilia^high^ ciliated cells (in addition to cilia components, also express the ion channel *BEST4*). We also recovered a very small population of cells we term “enteroendocrine cells,” based on unique expression of gastric inhibitory polypeptide (*GIP*), which is typically produced by intestinal and gastric enteroendocrine cells and *LGR5*, which classically marks stem cell populations in the gastrointestinal mucosa (Basak et al., 2017). Consistent with the use of nasal swabs for cell collection, we did not recover stromal cell populations such as endothelial cells, fibroblasts, or pericytes (Deprez et al., 2020; Garcıá et al., 2019; Ordovas-Montanes et al., 2018).

We similarly analyzed immune cell types for additional diversity. Among macrophages (coarse annotation), we resolved 5 distinct subtypes (Figure S3B). *FFAR4*^high^ macrophages are defined by expression of *FFAR4*, *MRC1*, *CHIT1*, and *SIGLEC11*, as well as chemotactic factors including *CCL18*, *CCL15*, genes involved in leukotriene synthesis (*ALOX5, ALOX5AP, LTA4H*), and toll-like receptors *TLR8* and *TLR2* (Figure S3F, full differentiating gene lists for immune subtypes found in Table S1). Interferon responsive macrophages are distinguished by elevated expression of anti-viral genes such as *IFIT3, IFIT2, ISG15,* and *MX1*, akin to the epithelial subsets labeled “interferon responsive,” along with *CXCL9*, *CXCL10*, *CXCL11*, which are likely indicative of IFNγ stimulation. *MSR1*^high^*C1QB*^high^ macrophages are defined by cathepsin expression (*CTSD, CTSL, CTSB*) and elevated expression of complement (*C1QB, C1QA, C1QC*), and lipid binding proteins (*APOE, APOC,* and *NPC2*). The fourth “specialized” subtype of macrophage we term “inflammatory macrophages,” which uniquely express inflammatory cytokines such as *CCL3, CCL3L1, IL1B, CXCL2,* and *CXCL3*. The remaining “*ITGAX*^high^” macrophages are distinguished from other immune cell types by *ITGAX*, *VCAN, PSAP, FTL, FTH1* and *CD163* (though these genes are shared by other specialized macrophages subsets). T cells are largely *CD69* and *CD8A* positive, with significantly-enriched expression of *ITGAE*, *ITGB7* and *CD44*, consistent with a T resident memory-like phenotype (Cosgrove et al., 2021), and we are not able to resolve a separate cluster of CD4 T cells (Table S1). Two specialized subtypes of CD8 T cells are annotated from this dataset: one defined by exceptionally high expression of early response genes (*FOSB, NR4A2,* and *CCL5*), and the other termed “interferon responsive cytotoxic CD8 T cells,” defined by granzyme and perforin expression (*GZMB, GZMA, GNLY, PRF1, GZMH*), anti-viral genes (*ISG20, IFIT3, APOBEC3C, GBP5*) and genes associated with effector CD8 T cell function (*LAG3, IL2RB, IKZF3, TBX21*). Multiple cell types could not be further subdivided from their coarse annotation (Figure 1B, Figure S3A-S3E), including mast cells, plasmacytoid DCs, B cells, and dendritic cells.

#### RNA Velocity and Pseudotemporal Ordering of Epithelial Cells

RNA velocity was modeled using the scVelo package, version 0.2.3 (Bergen et al., 2020; La Manno et al., 2018). Briefly, RNA velocity analysis leverages the dynamic relationships between expression of unspliced (intron-containing) and spliced (exonic) RNA across thousands of variable genes, enabling 1) estimation of the directionality of transitions between distinct cells and cell types, and 2) identification of putative driver genes behind these transitions. Using cluster annotations previously assigned from iterative clustering in Seurat, cells from epithelial cell types were pre-processed according to the scVelo pipeline: genes were normalized using default parameters (pp.filter_and_normalize), principal components and nearest neighbors in PCA space were calculated (using defaults of 30 PCs, 30 nearest neighbors), and the first and second order moments of nearest neighbors were computed, which are used as inputs into velocity estimates (pp.moments). RNA velocity was estimated using the scVelo tool tl.recover_dynamics with default input parameters, which maps the full splicing kinetics for all genes and tl.velocity, with mode = 'dynamical'. Top velocity transition “driver” genes were identified by high “fit_likelihood” parameters from the dynamical model, and are used for visualization in Figure S2L. The same approaches were used for modeling RNA velocity among only basal, secretory, and goblet cells (Figures 2F–2I), only ciliated cells (Figures 2K–2N), and only COVID-19 or only Control cells (Figures 2P, 2Q). For RNA velocity analysis of ciliated cells or basal, secretory and goblet cells, the velocity pseudotime was calculated using the tl.velocity_pseudotime function with default settings.

#### Metatranscriptomic Classification of Reads from Single-Cell RNA-Seq

ScRNA-seq protocols utilize poly-adenylated RNA capture and reverse transcription to generate snapshots of the transcriptional status of each individual cell. As several pathogens and commensal microbes also utilize poly-adenylation for RNA intermediates, or contain poly-adenylated stretches of RNA within their genomes, they may also be represented within scRNA-seq libraries. To identify co-detected microbial taxa present in the cell-associated or ambient RNA of nasopharyngeal swabs, we used the Kraken2 software implemented using the Broad Institute viral-ngs pipelines on Terra (https://github.com/broadinstitute/viral-pipelines/tree/master) (Wood et al., 2019). A previously-published reference database included human, archaea, bacteria, plasmid, viral, fungi, and protozoa species and was constructed on May 5, 2020, therefore included sequences belonging to the novel SARS-CoV-2 virus (Lemieux et al., 2021). Inputs to Kraken2 were: kraken2_db_tgz = ”gs://pathogen-public-dbs/v1/kraken2-broad-20200505.tar.zst,” krona_taxonomy_db_kraken2_tgz = ”gs://pathogen-public-dbs/v1/krona.taxonomy-20200505.tab.zst,” ncbi_taxdump_tgz = ”gs://pathogen-public-dbs/v1/taxdump-20200505.tar.gz,” trim_clip_db = ”gs://pathogen-public-dbs/v0/contaminants.clip_db.fasta” and spikein_db = ”gs://pathogen-public-dbs/v0/ERCC_96_nopolyA.fasta.” Viral species with fewer than 5 reads were considered spurious and excluded. Swabs from two individuals contain rare reads classified as Influenza A virus species, and we observe no evidence for other seasonal human coronaviruses, Influenza B virus, metapneumovirus, or orthopneumovirus. Swabs from two individuals with mild/moderate COVID-19 contain exceptionally high abundances of reads classified as Rhinovirus A (2.1e5 and 2.4e5 reads) (Figure 4A).

#### Correction for Ambient Viral RNA and Defining High-Confidence SARS-CoV-2 RNA^+^ Cells

Data from high-throughput scRNA-seq platforms frequently experience low-levels of non-specific RNA assigned to cell barcodes that does not represent true cell-derived transcriptomic material, but rather contamination from the ambient pool of RNA. To safeguard against spurious assignment of SARS-CoV-2 RNA to cells without true intracellular viral material, i.e., viral RNA non-specifically picked up from the microenvironment as a component of ambient RNA contamination, we employed the following corrections and statistical tests to control for ambient viral RNA and enable confident assignments for SARS-CoV-2 RNA^+^ cells. Similar to approaches previously described, we tested whether the abundance of viral RNA within a given single cell was significantly higher than expected by chance given the estimate of ambient RNA contaminating that cell, as well as the proportion of viral RNA of the total ambient RNA pool (Cao et al., 2020; Delorey et al., 2021; Fleming et al., 2019; Kotliar et al., 2020). First, this required modeling and estimating the ambient RNA fraction associated with each individual swab. Here, we employed CellBender (https://github.com/broadinstitute/CellBender), a software package built to learn the ambient RNA profile per sample and provide an ambient RNA-corrected output (Fleming et al., 2019). Input UMI count matrices contained the top 10,000 cell barcodes, therefore including at least 70% cell barcodes sampling the ambient RNA and low-complexity cell barcodes. CellBender’s remove-background function was run with default parameters and–fpr 0.01–expected-cells 500–low-count-threshold 5. Using the corrected output from each sample’s count matrix following CellBender, we calculated the proportion of ambient contamination per high-quality cell by comparing to the single-cell’s transcriptome pre-correction, and summed all UMI from background cell barcodes to recover an estimate of the total ambient pool. Next, we tested whether the abundance of viral RNA in a given single cell was significantly above the null abundance given the ambient RNA characteristics using an exact binomial test (implemented in R: binom.test):

$$\begin{array}{l} P\left(x\right)=\frac{n!}{\left(n-x\right)!x!}\;p^{x}q^{n-x}\;\text{where x }=\text{ SARS-CoV-2 UMI per cell, n }=\text{ total UMI per cell}\\ \text{p }=\left(\text{ambient fraction per cell}\right)\text{×}\left(\text{SARS-CoV-2 UMI fraction of all ambient UMI}\right)\text{, and q }=\text{ 1}-\text{p} \end{array}$$

P values were FDR-corrected within sample, and cells whose SARS-CoV-2 UMI abundance with FDR < 0.01 were considered “SARS-CoV-2 RNA+.”

During SARS-CoV-2 infection, viral uncoating from endosomal vesicles releases the positive, single-stranded, 5′ capped, poly-adenylated genome into the host cytosol (Figures 4F, 4G). Here, translation of non-structural proteins proceeds first by templating directly off of the viral genome, generating a replication and transcription complex. The viral replication complex then produces both 1) negative strand genomic RNA intermediates, which serve as templates for further positive strand genomic RNA and 2) nested subgenomic mRNAs which are constructed from a 5′ leader sequence fused to a 3′ sequence encoding structural proteins for production of viral progeny (e.g., Spike, Envelope, Membrane, Nucleocapsid). Generation of nested subgenomic mRNAs relies on discontinuous transcription occurring between pairs of 6-mer transcriptional regulatory sequences (TRS), one 3′ to the leader sequence (termed leader TRS, or TRS-L), and others 5′ to each gene coding sequence (termed body TRS, or TRS-B) (Sawicki et al., 2007). We reasoned that SARS-CoV-2 aligning UMI could be readily distinguished by their strandedness (aligning to the negative versus positive strand) and whether they fell within coding regions, across intact TRS (indicating RNA splicing had not occurred for that RNA molecule at that splice site) or across a TRS with leader-to-body fusions (corresponding to subgenomic RNA, Figure 4F, 4G, Figure S6A). Notably, single cells containing reads aligning to spliced (subgenomic) RNA are heavily skewed toward those cells that contain the highest overall abundances of viral UMI – this may be an accurate reflection of coronavirus biology, wherein subgenomic RNA are most frequent within cells robustly producing new virions and total viral genomic material, but also points to inherent limitations in the detection of low-frequency RNA species by single-cell RNA-seq technologies.

#### Differential Expression by Group, Cell Type, or Viral RNA Status

To compare gene expression between cells from distinct disease groups we employed a likelihood ratio test assuming a negative binomial distribution. Cells from each cell type belonging to either COVID-19 WHO 1-5 (mild/moderate), COVID-19 WHO 6-8 (severe), or Control WHO 0 were compared in a pairwise manner, implemented using the Seurat FindAllMarkers function (test.use = “negbinom”). We considered genes as differentially expressed with an FDR-adjusted p value < 0.001 and log fold change > 0.25. To compare gene expression between SARS-CoV-2 RNA^+^ cells and bystander cells (from COVID-19 participants, but without intracellular viral RNA) we used a negative binomial generalized linear model implemented using DESeq2 (Love et al., 2014). Here, we employed the following criteria for SARS-CoV-2 RNA^+^ versus bystander testing: 1) we only tested cell types containing at least 15 SARS-CoV-2 RNA^+^ cells, 2) for each cell type, we restricted our bystander cells to the same participants as the SARS-CoV-2 RNA^+^ cells, 3) in comparisons where bystander cells were substantially more numerous than SARS-CoV-2 RNA^+^ cells, we randomly sub-sampled the bystander cells to at most 4x the number of SARS-CoV-2 RNA^+^ cells, and 4) we ensured that the sampled bystander cells for comparison matched the cell quality distribution of the SARS-CoV-2 RNA^+^ cells, based on binned deciles of UMI/cell. DESeq2 was run with default parameters and test = “Wald.” Gene ontology analysis was run using the Database for Annotation, Visualization, and Integrated Discovery (DAVID) (Huang et al., 2009). Gene set enrichment analysis (GSEA) was completed using the R package fgsea over genes ranked by average log foldchange expression between each group, including all genes with an average expression > 0.5 UMI within each respective cell type (Korotkevich et al., 2021). Gene lists corresponding to “Shared IFN Response,” “Type I IFN Specific Response” and “Type II IFN Specific Response” are derived from previously-published population RNA-seq data from nasal epithelial basal cells treated *in vitro* with 0.1 ng/mL – 10 ng/mL IFNα or IFNγ for 12 h (Ziegler et al., 2020). Module scores were calculated using the Seurat function AddModuleScore with default inputs.

#### Statistical Testing

All statistical tests were implemented either in R (v4.0.2) or Prism (v6) software (R Core Team, 2019). Comparisons between cell type proportions by disease group were tested using a Kruskal-Wallis test with FDR correction across all cell types, implemented in R using the kruskal.test, and p.adjust functions. Post-tests for between-group pairwise comparisons used Dunn’s test. Spearman correlation was used where appropriate, implemented using the cor.test function in R. All testing for differential expression was implemented in R using either Seurat, scVelo, or DESeq2, and all results were FDR-corrected as noted in specific STAR Methods sections. P values, n, and all summary statistics are provided either in the results section, figure legends, figure panels, or supplementary tables. Prism (v6), R (v4.0.2) packages ggplot2 (v3.3.2 (Wickham, 2016)), Seurat (v3.2.2 (Butler et al., 2018)), ComplexHeatmap (v2.7.3 (Gu et al., 2016)), Circlize (0.4.11 (Gu et al., 2014)), fgsea (v.1.16.0 (Korotkevich et al., 2021)), DESeq2 (v1.30.0 (Love et al., 2014)), and Python (v3.8.3) package scVelo (v0.3.0 (Bergen et al., 2020)) were used for visualization.

## Data Availability

• Single-cell RNA-seq data is publicly available for download and visualization via the Single Cell Portal: https://singlecell.broadinstitute.org/single_cell/study/SCP1289/. This paper also analyzes existing, publicly available data. Accession numbers and links are listed in the key resources table. Interim data was also deposited in a single-cell data resource for COVID-19 studies: https://www.covid19cellatlas.org (Ballestar et al., 2020). Custom reference FASTA and GTF for SARS-CoV-2 is available for download: https://github.com/ShalekLab/SARSCoV2-genome-reference. Additional Supplemental Items are available from Mendeley Data at https://doi.org/10.17632/pjr7b8sbf8.1. • All original code has been deposited at GitHub (https://github.com/ShalekLab) and is publicly available as of the date of publication. • Any additional information required to reanalyze the data reported in this paper is available from the lead contact upon request

## Supplemental information

Table S1. Cell type marker genes, related to Figures 1, 2, and S3

Table S2. Differentially expressed genes between cell types from control WHO 0 versus COVID-19 WHO 1–5 (mild/moderate), related to Figure 3

Table S3. Differentially expressed genes between cell types from control WHO 0 versus COVID-19 WHO 6–8 (severe), related to Figure 3

Table S4. Differentially expressed genes between cell types from COVID-19 WHO 1–5 (mild/moderate) versus COVID-19 WHO 6–8 (severe), related to Figure 3

Table S5. Common differentially expressed genes between SARS-CoV-2 RNA^+^ cells and bystander cells, related to Figure 6
